# A study of fracture mechanics for compact tensile specimen of Al6061-SiC metal matrix composite

**DOI:** 10.1038/s41598-025-89041-w

**Published:** 2025-02-17

**Authors:** Ehab Samir Mohamed Mohamed Soliman

**Affiliations:** Mechatronics and Robotics Engineering Department, Faculty of Engineering, Egyptian Russian University, Badr City, Cairo 11829 Egypt

**Keywords:** Mode I stress intensity factor, Crack, T-stress, Compact tension CT specimen, Al6061-SiC metal matrix composite, Mechanical engineering, Composites

## Abstract

The main contribution of the present work is the display of the impact of the addition of SiC into the aluminum alloy Al6061. For this reason, the Mode I stress intensity factor K_I_ and T-stress for compact tension CT specimen are evaluated using 3D finite element analysis (FEA). The material used here in the compact tension CT specimen is Al6061-SiC metal matrix composites reinforced with various volume fractions of 4%, 6%, 10%, 12%, and 14% of SiC particles. Three different crack lengths (a/H) ratios of 0.35, 0.43, and 0.5 are considered through the analysis. Only half of the model of the cracked compact tension CT specimen with a subjected load of a magnitude *P* = 603 N is analyzed, and K_I_, T_11_-stress, and T_33_-stress are computed. From the FEA results, it is observed that the K_I_, T_11_-stress, and T_33_-stress are mainly influenced by the volume fractions of reinforced SiC particles. A more significant decrease in the values of K_I_, T_11_-stress, and T_33_-stress is found in the Al6061-14vol.%SiC composite CT specimen. Where FEA results of K_I_ for the Al6061-14vol.%SiC composite CT specimen exhibited reduction percentages of 5.4%, 5.6%, and 5.7%, respectively, for (a/H) = 0.35, 0.43, and 0.5, as compared to those of Al6061. FEA values of T_11_-stress for the Al6061-14vol.%SiC composite CT specimen reduced by 5.5%, 5.6%, and 5.7%, respectively, for (a/H) = 0.35, 0.43, and 0.5, respectively, over those of Al6061. Also, the decrement percentages of FEA results of T_33_-stress for the Al6061-14vol.%SiC composite CT specimen over those of Al6061 were found to be 17.1%, 16.6%, and 16.5%, respectively, for (a/H) = 0.35, 0.43, and 0.5, respectively. Overall, fracture mechanics properties are improved by the addition of SiC particulates into the Al6061 alloy.

## Introduction

Fracture mechanics is concerned with the study of the load-bearing capacity of a body, including the initial cracks and various laws for the growth of cracks^1^. Cracks/notches may exist under complex loading in built-up welded structures, such as ships, aircraft, building frames, bridge decks, oil refineries, and offshore oil platform conditions; therefore, investigation of crack initiation and propagation is very important^2^. A fracture criterion can be based on the near-tip strain or crack-tip contraction^3^. When a stress field near the crack tip in a structure approaches the same value as in a test specimen under a fracture load, it assumes a fracture is occurring^4^. Studies on the crack in a flat plate cover many engineering applications where the area around the crack tip in the majority parts can be considered as plate form, such as cracks in a tube whose length is smaller than its diameter^1^. Fracture toughness of the material is defined as the resistance of a material to fracture, and there are many factors that affect fracture toughness, such as composition, load, microstructure, geometry, and temperature^5^. To calculate the stress intensity factors (SIFs), many methods have been developed, and among these is the finite element method (FEM), which is the most popular and gives a reasonably accurate result^6^. For linear elastic fracture mechanics problems, the finite element method is a suitable approach to evaluating the stress intensity factor^7^. In finite element analysis, to estimate fracture parameters, such as stress intensity factor and strain energy release rate, a refined mesh or singular elements must be generated around the crack tip^2^. Most commonly used lightweight materials, i.e., aluminum (Al) alloys, have outstanding formability and can be processed in different methods^8^. Metal-matrix composites (MMCs) are heterogeneous materials consisting of a metallic matrix and (typically ceramic) reinforcements, and these materials are used in many applications such as automotive engineering, the aerospace industry, and the defense industry^9^. Contrary to homogeneous alloys and resin matrix composites, MMC has the following advantages: higher strength and stiffness values, a lower coefficient of thermal expansion, and high temperature resistance^10^. Composite materials are used in many applications such as aerospace, maritime, electronic, and medical fields where the reinforcements such as alumina (Al_2_O_3_), silicon carbide (SiC), and boron carbide (B_4_C) improve the properties without affecting the original properties of the base metal^11^. Over the last three decades, aluminum-based metal matrix composites have increasingly replaced the traditional engineering materials^12^. SiC, Al_2_O_3_, B_4_C, and graphite are common reinforcements for aluminum matrices^12^. Silicon carbide (SiC) is a ceramic material, and its particles are used as reinforcement in Al6061-SiC composites, where aluminum 6061 is considered a matrix in Al6061-SiC composites^13^. SiC is widely used in the construction and machinery industries, and its advantages are wear resistance, high hardness, low density, anti-corrosive properties, and high thermal conductivity^14^. Doddamani and Kaleemulla^15^ prepared compact tension specimens for different crack length to width (a/w) ratios according to ASTM E-399, and these specimens were made from aluminum 6061-graphite with 3, 6, 9, and 12% graphite particles. They used these specimens to determine fracture toughness by crack length and critical load. They inferred that Al6061-9% Gr for a/w = 0.45 has the maximum fracture toughness of 16.74 MPa √m. Biradar and Savanur^16^ carried out finite element analysis (FEA) of a compact tension specimen for fracture-toughness evaluation and showed that FEA provides reliable J-integral values with satisfactory precision. Raviraj et al.^17^ machined the compact tension specimens made from aluminum (Al6061) matrix reinforced with 3 wt%, 5 wt%, and 7 wt% TiC particles according to ASTM E399 to determine the fracture toughness. The stress intensity factors SIFs for CT specimens of various ratios of thickness to width B/W were experimentally estimated, and the calculated SIFs were plotted against various B/W ratios for various Al6061-TiC composites. They noticed the influence of the contrast of TiC reinforcement particles with the Al6061 alloy matrix on the fracture toughness of the material, where the fracture toughness of Al6061-TiC (3 wt%–7 wt%) metal matrix composites varied between 16.4 and 19.2 MPa√m. Kudari and Kodancha^18^ computed stress intensity factor and T-stress for interstitial free (IF) steel CT specimens for different specimen thicknesses (B/W = 0.1-1) and crack length-to-width ratios (a/W = 0.45–0.7) using 3D elastic finite element analysis FEA, software ABAQUS 6.5. They found that a polynomial equation of third order fits the 3D FEA results with excellent agreement and expressed the relation between K_I−max_, a/W, and σ as the following^18^:

1 $$\frac{{K_{I - \max } }}{{\sigma \sqrt {\pi a} }} = 4.48287 - 14.99985\left( \frac{a}{W} \right) + 20.44016\left( \frac{a}{W} \right)^{2} - 3.85185\left( \frac{a}{W} \right)^{3}$$

Where K_I−max_ is the maximum stress intensity factor, σ is the applied stress, W is the width of the specimen, and a is the crack length.

Guddhur et al.^19^ conducted fracture toughness testing for Al-SiCp compact tension specimen using the standard universal testing machine. In their study, they used two techniques, Taguchi’s and ANOVA, to investigate the process parameters that influence the fracture toughness of aluminum-silicon carbide particulate. Taguchi’s analysis showed a decrease in the composite’s load-carrying capacity with an increase in the a/W ratio. The ANOVA analysis revealed that the effect of composition and a/W ratio on the fracture toughness is greater than the grain size of the SiC. In this background, the investigation of aluminum-silicon carbide particulate composites in the field of fracture mechanics is considered. Many researchers worked on investigation of the fracture behavior of compact tension CT specimen made from Al–SiC reinforced Metal matrix composites (MMC) and compared the results with those of made from Al alloy. Their studies depended on determination of mode I stress intensity factor or fracture toughness. However, work related to the combination of evaluation mode I stress intensity factor, T_11_-stress, and T_33_-stress besides to calculate the mechanical properties for Al–SiC reinforced Metal matrix composites, is rare and considers as comprehensive work to give more details about influence of the adding SiC particles into Al alloy. In this manuscript, an endeavor has been made to art more specifics about the influence of the addition of SiC into the aluminum alloy Al6061. Finite element three-dimensional analysis FEA is carried out for Al6061-SiC composite compact tension specimen with various volume fractions of SiC. The magnitudes of Mode I stress intensity factor, T_11_-stress, and T_33_-stress for CT specimen have been extracted. FEA results are investigated to quantify the effects of the addition SiC particles to the aluminum alloy Al6061.

## Theoretical background

**Fig. 1:**
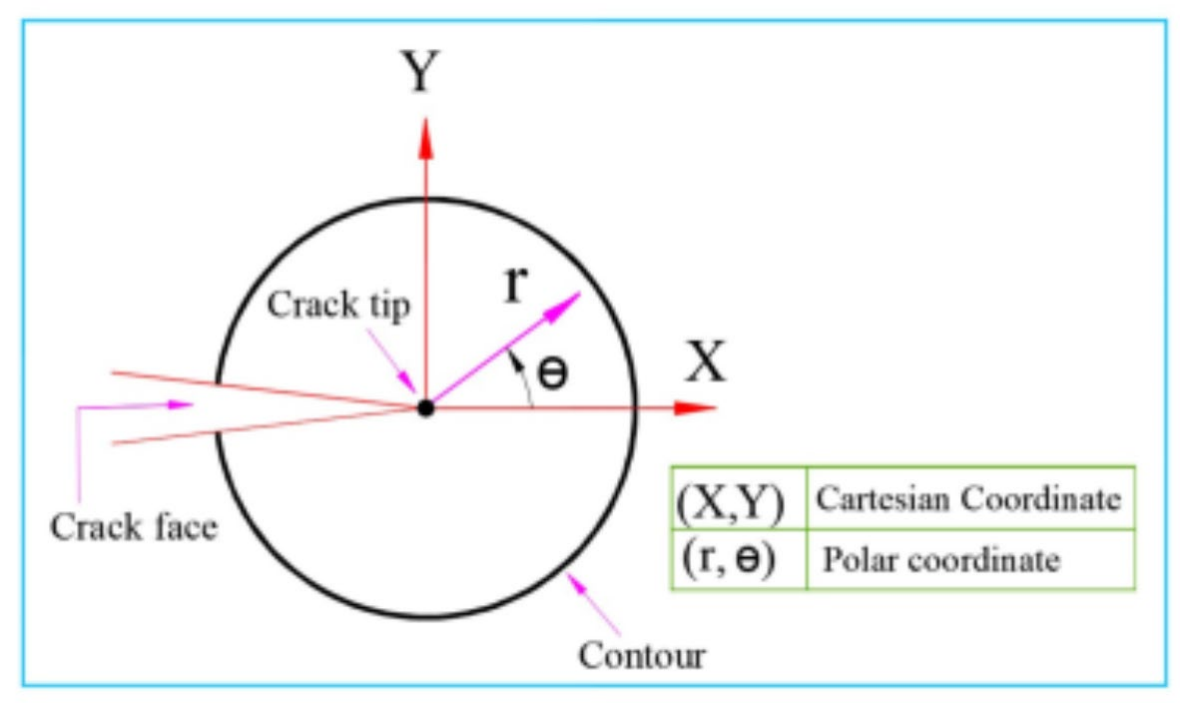
Co-ordinate systems of crack tip.

The co-ordinate systems and contour around the crack tip shown in Fig. 1 are used in this study to describe stresses near the crack tip.

In case of plane strain condition, the equations of displacement fields near the crack tip for a cracked body originally found in^20^ and can be written as^21^:

2 $$\begin{gathered} u_{x} = \frac{{K_{I} }}{4G}\sqrt {\frac{r}{2\pi }} \left[ {\left( {5 - 8\nu } \right)\cos \frac{\theta }{2} - \cos \frac{3\theta }{2}} \right] \hfill \\ \, + \frac{{K_{II} }}{4G}\sqrt {\frac{r}{2\pi }} \left[ {\left( {9 - 8\nu } \right)\sin \frac{\theta }{2} + \sin \frac{3\theta }{2}} \right] \hfill \\ \, + \left( {1 - \nu^{2} } \right)\frac{r}{E}\left( {T\cos \theta - 4B_{n} \sin \theta } \right) \hfill \\ \, + O\left( {r^{{{3 \mathord{\left/ {\vphantom {3 2}} \right. \kern-0pt} 2}}} } \right) \hfill \\ \end{gathered}$$

3 $$\begin{gathered} u_{y} = \frac{{K_{I} }}{4G}\sqrt {\frac{r}{2\pi }} \left[ {\left( {7 - 8\nu } \right)\sin \frac{\theta }{2} - \sin \frac{3\theta }{2}} \right] \hfill \\ \, - \frac{{K_{II} }}{4G}\sqrt {\frac{r}{2\pi }} \left[ {\left( {3 - 8\nu } \right)\cos \frac{\theta }{2} + \cos \frac{3\theta }{2}} \right] \hfill \\ \, - \left( {1 + \nu } \right)\frac{r}{E}\left( {\nu T\sin \theta + 4\left( {1 - \nu } \right)B_{n} \cos \theta } \right) \hfill \\ \, + O\left( {r^{{{3 \mathord{\left/ {\vphantom {3 2}} \right. \kern-0pt} 2}}} } \right) \hfill \\ \end{gathered}$$

Where: u_x_ and u_y_ are displacement fields in the case of plane strain condition; B_n_ is the coefficient related to Williams’ expansion in anti-symmetric mode; K_I_ and K_II_ are the mode I and mode II stress intensity factors; E is Young’s modulus; ν is Poisson’s ratio; G is the shear modulus; T is a constant acting parallel to the crack surfaces and (r, θ) is the polar coordinate.

Assuming the plastic zone nearby the crack tip is very small, i.e. r→0 consequently it can be written the Westergaard’s complex functions Z and Z’ as the following^22^:

4 $$Z = \sum\limits_{m = 0}^{M} {A_{m} } r^{{m - {1 \mathord{\left/ {\vphantom {1 2}} \right. \kern-0pt} 2}}} \left[ {\cos \left( {m - \frac{1}{2}} \right)\theta + i\sin \left( {m - \frac{1}{2}} \right)\theta } \right]$$

5 $$Z^{\prime} = \frac{dZ}{{dz}} = \sum\limits_{m = 0}^{M} {\left( {m - \frac{1}{2}} \right)A_{m} } r^{{m - {3 \mathord{\left/ {\vphantom {3 2}} \right. \kern-0pt} 2}}} \left[ {\cos \left( {m - \frac{3}{2}} \right)\theta + i\sin \left( {m - \frac{3}{2}} \right)\theta } \right]$$

6 $$z = x + iy = re^{iN\theta }$$

7 $$y = r\sin \theta = 2r\sin \frac{\theta }{2}\cos \frac{\theta }{2}$$

From the above equations Eqs. (3, 4), the real and imaginary parts can be got as^22^:

8 $${\text{Re}} Z = \sum\limits_{m = 0}^{M} {A_{m} } r^{{m - {1 \mathord{\left/ {\vphantom {1 2}} \right. \kern-0pt} 2}}} \cos \left( {m - \frac{1}{2}} \right)\theta$$

9 $${\text{Re}} Z^{\prime} = \sum\limits_{m = 0}^{M} {\left( {m - \frac{1}{2}} \right)A_{m} } r^{{m - {3 \mathord{\left/ {\vphantom {3 2}} \right. \kern-0pt} 2}}} \cos \left( {m - \frac{3}{2}} \right)\theta$$

10 $${\text{Im}} Z = \sum\limits_{m = 0}^{M} {A_{m} } r^{{m - {1 \mathord{\left/ {\vphantom {1 2}} \right. \kern-0pt} 2}}} \sin \left( {m - \frac{1}{2}} \right)\theta$$

11 $${\text{Im}} Z^{\prime} = \sum\limits_{m = 0}^{M} {\left( {m - \frac{1}{2}} \right)A_{m} } r^{{m - {3 \mathord{\left/ {\vphantom {3 2}} \right. \kern-0pt} 2}}} \sin \left( {m - \frac{3}{2}} \right)\theta$$

The stresses in complex form can be expressed as^22^:

12 $$\sigma_{xx} = {\text{Re}} Z - y{\text{Im}} Z^{\prime}$$

13 $$\sigma_{yy} = {\text{Re}} Z + y{\text{Im}} Z^{\prime}$$

14 $$\tau_{xy} = - y{\text{Re}} Z^{\prime}$$

Where: σ_xx_ is the stress in the x-direction, σ_yy_ is the stress in the y-direction and τ_xy_ is the shear stress.

For mode I, the first order stress field equations can be written as^22^:

15 $$\left[ \begin{gathered} \sigma_{xx} \hfill \\ \sigma_{yy} \hfill \\ \tau_{xy} \hfill \\ \end{gathered} \right] = \frac{{K_{I} }}{{\sqrt {2\pi r} }}\cos \frac{\theta }{2}\left[ \begin{gathered} 1 - \sin \frac{\theta }{2}\sin \frac{3\theta }{2} \hfill \\ 1 + \sin \frac{\theta }{2}\sin \frac{3\theta }{2} \hfill \\ \sin \frac{\theta }{2}\cos \frac{3\theta }{2} \hfill \\ \end{gathered} \right]$$

The stress in the z-direction originally introduced in^22^ and can be expressed as^23^:

16 $$\sigma_{zz} = \left\{ \begin{gathered} \nu \left( {\sigma_{xx} + \sigma_{yy} } \right) \, = \frac{{2\nu K_{I} }}{{\sqrt {2\pi r} }}{\text{cos}}\frac{\theta }{{2}}{\text{ (for plane strain)}} \hfill \\ {\text{0 (for plane stress)}} \hfill \\ \end{gathered} \right.$$

The plastic zone radius r(θ) as a function of the polar angle θ, can be expressed as^24^:

17 $$\left\{ \begin{gathered} r\left( \theta \right) = \frac{{K_{I}^{2} }}{{2\pi \sigma_{y}^{2} }}\cos^{2} \frac{\theta }{2}\left[ {3\sin^{2} \frac{\theta }{2} + 1} \right]{ ; }\left( {\text{for plane stress}} \right) \hfill \\ r\left( \theta \right) = \frac{{K_{I}^{2} }}{{2\pi \sigma_{y}^{2} }}\cos^{2} \frac{\theta }{2}\left[ {3\sin^{2} \frac{\theta }{2} + \left( {1 - 2\nu } \right)^{2} } \right]{ ; }\left( {\text{for plane strain}} \right) \hfill \\ \end{gathered} \right.$$

For standard compact tension metallic specimens, according to the ASTM test for plane strain fracture toughness, the stress intensity factor originally introduced by Duggan et al.^25^ and can be written as^26^:

18 $$K_{I} = \frac{P}{B\sqrt H }\left[ {29.6\left( \frac{a}{H} \right)^{\frac{1}{2}} - 185.5\left( \frac{a}{H} \right)^{\frac{3}{2}} + 655.7\left( \frac{a}{H} \right)^{\frac{5}{2}} - 1017\left( \frac{a}{H} \right)^{\frac{7}{2}} + 638.9\left( \frac{a}{H} \right)^{\frac{9}{2}} } \right]$$

Energy-release rate G_I_ can be written as^27^:

19 $$G_{I} = \frac{{K_{I}^{2} }}{{E^{\prime}}}$$

20 $$E^{\prime} = \left\{ \begin{gathered} E;{\text{ (for plane stress)}} \hfill \\ {E \mathord{\left/ {\vphantom {E {\left( {1 - \nu^{2} } \right)}}} \right. \kern-0pt} {\left( {1 - \nu^{2} } \right)}};{\text{ (for plane strain)}} \hfill \\ \end{gathered} \right.$$

Where: K_I_ is the mode I stress intensity factors; E is Young’s modulus and ν is Poisson’s ratio.

### Fracture model and mesh convergence

**Fig. 2:**
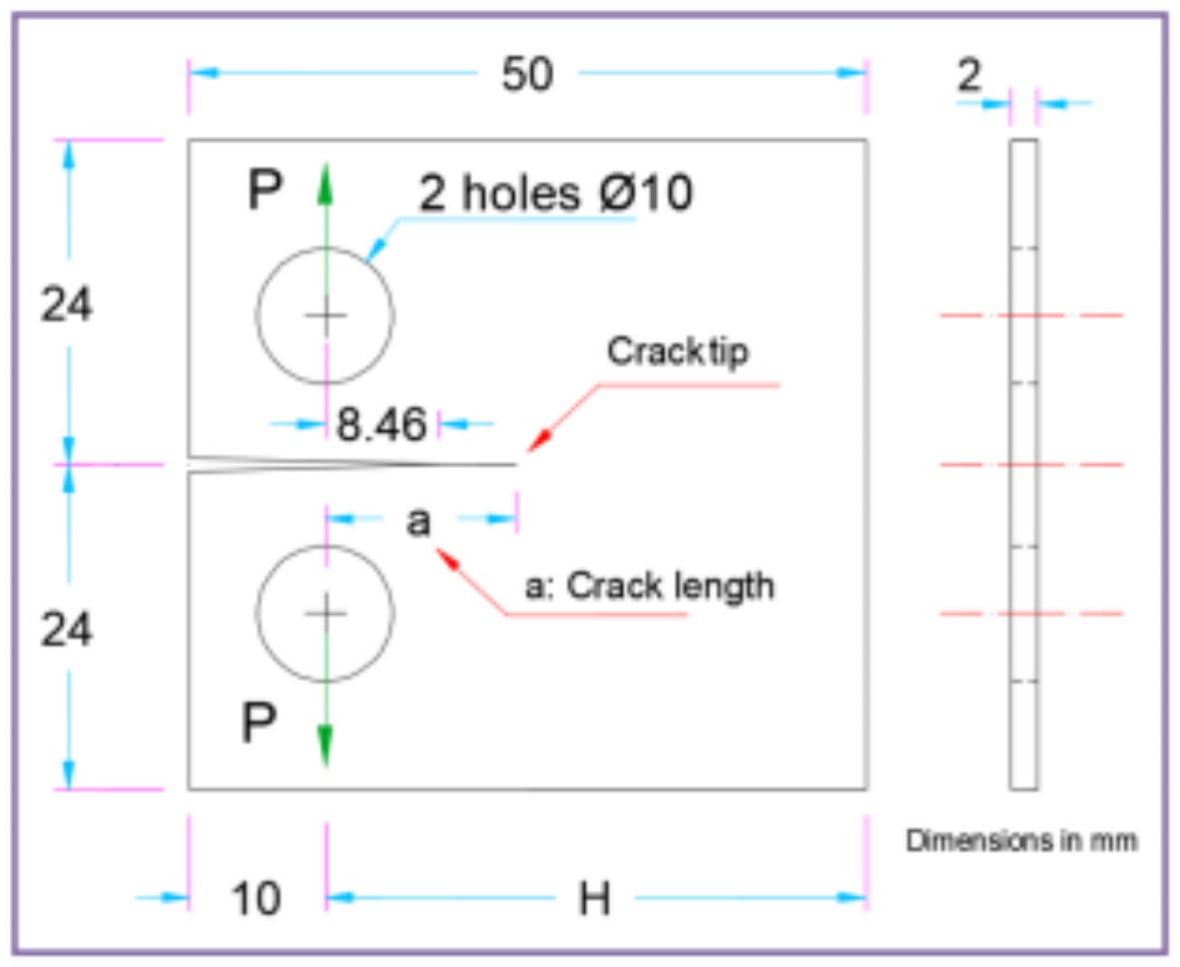
Geometry of the compact tension CT specimen.

**Fig. 3:**
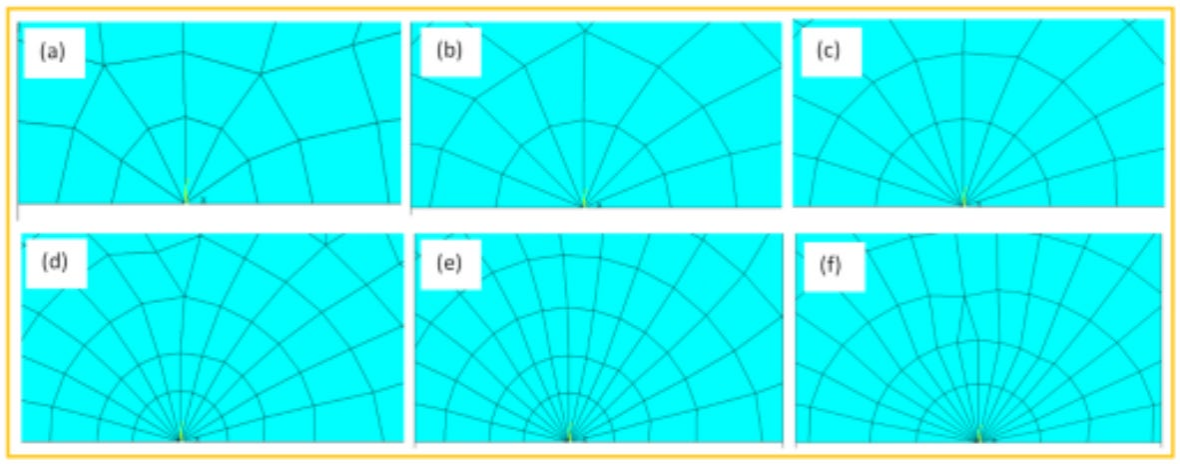
Mesh around the crack tip: (a) 6 singular elements; (b) 8 singular elements; (c) 10 singular elements; (d) 12 singular elements; (e) 14 singular elements; (f) 16 singular elements.

**Fig. 4:**
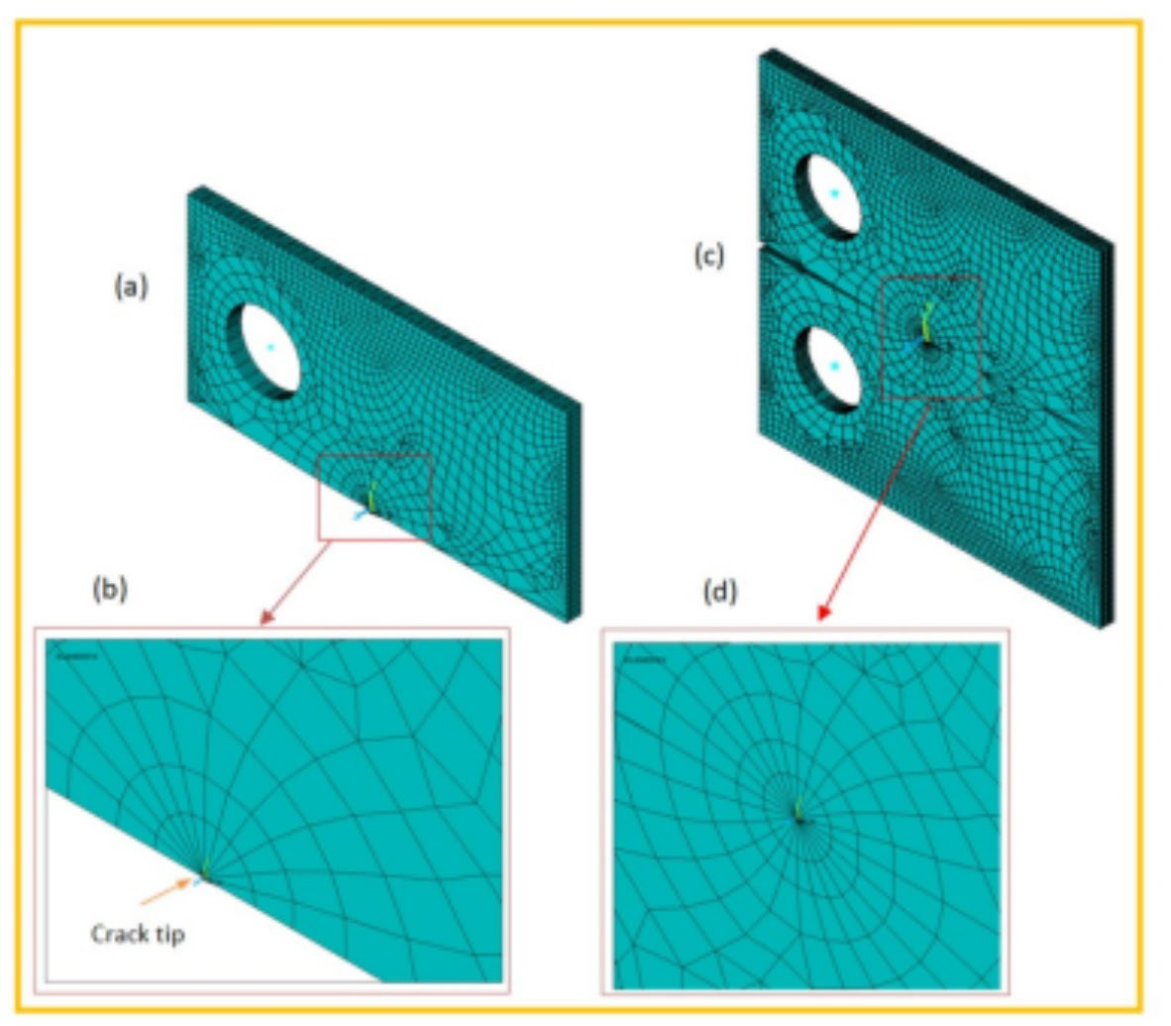
FE mesh of the half compact tension CT specimen: (a) half compact tension CT specimen; (b) singular elements around crack tip; (c) reflect about XZ of cyclic symmetry; (d) singular elements around crack tip with reflect about XZ.

**Fig. 5:**
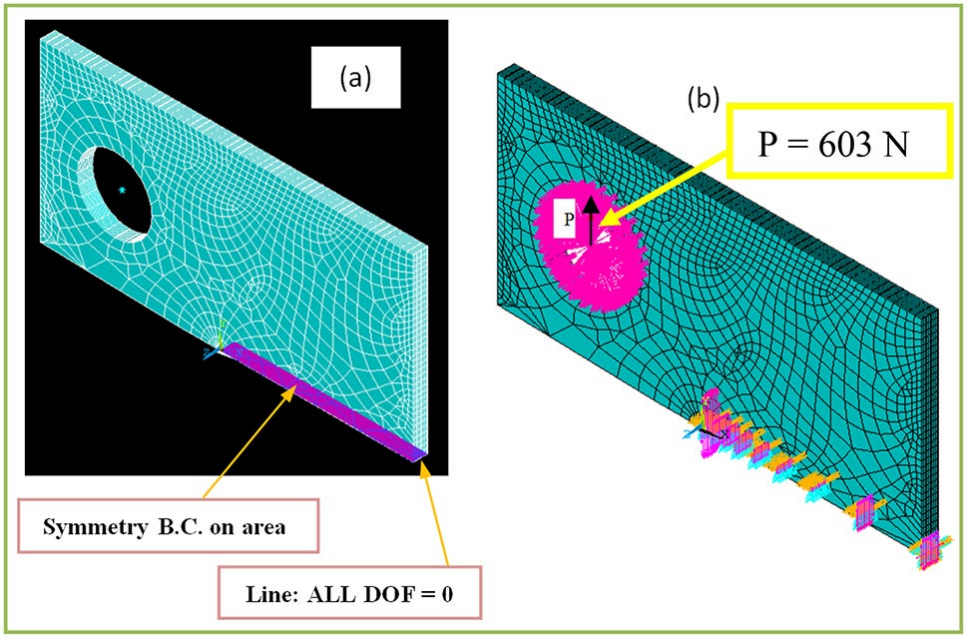
Boundary conditions of half compact tension CT specimen: (a) symmetry boundary conditions; (b) point of imposed applied load.

**Fig. 6:**
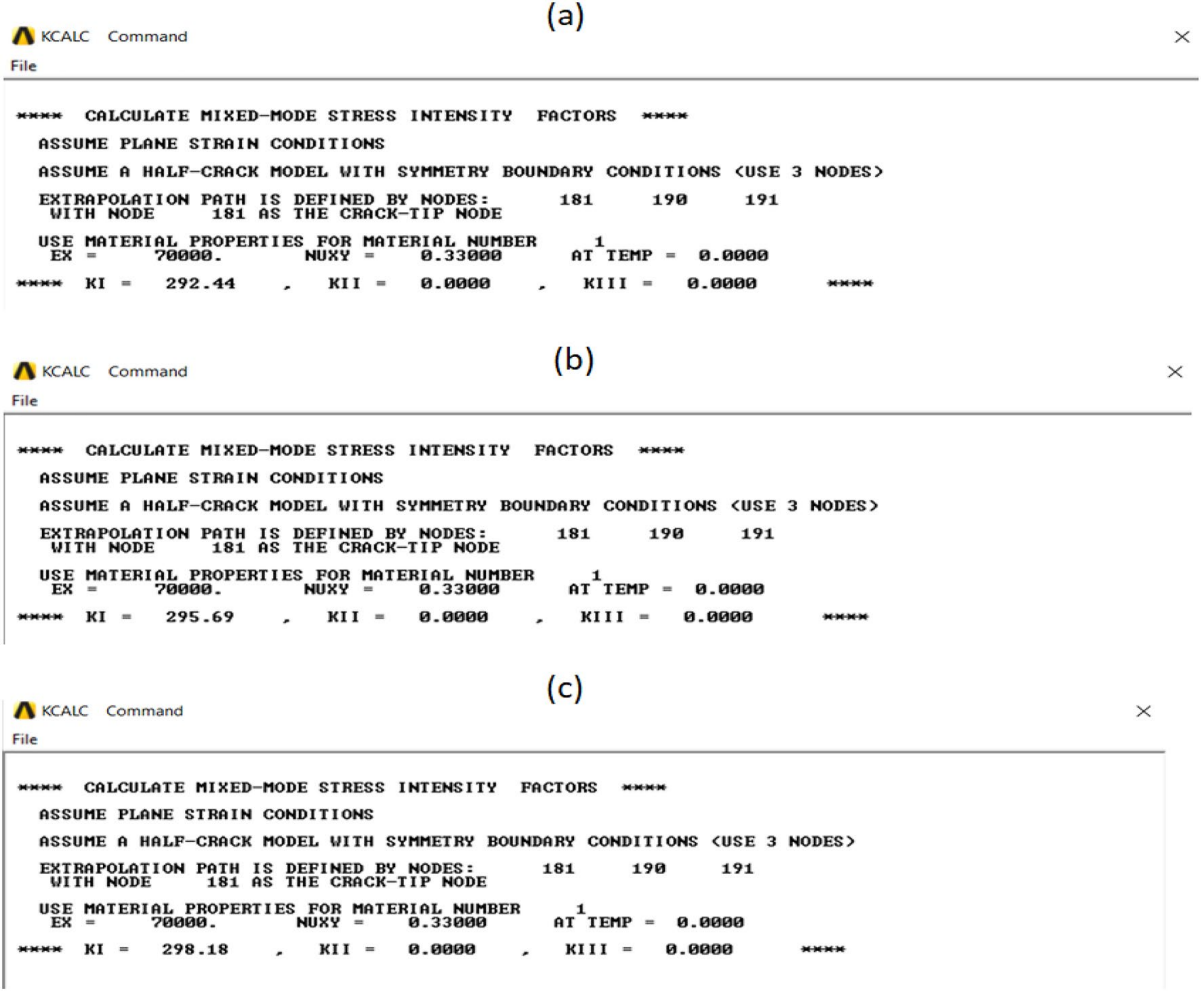
Mode I stress intensity factor (KI) with different number of singular elements: (a) 6 singular elements; (b) 8 singular elements; (c) 10 singular elements.

**Fig. 7:**
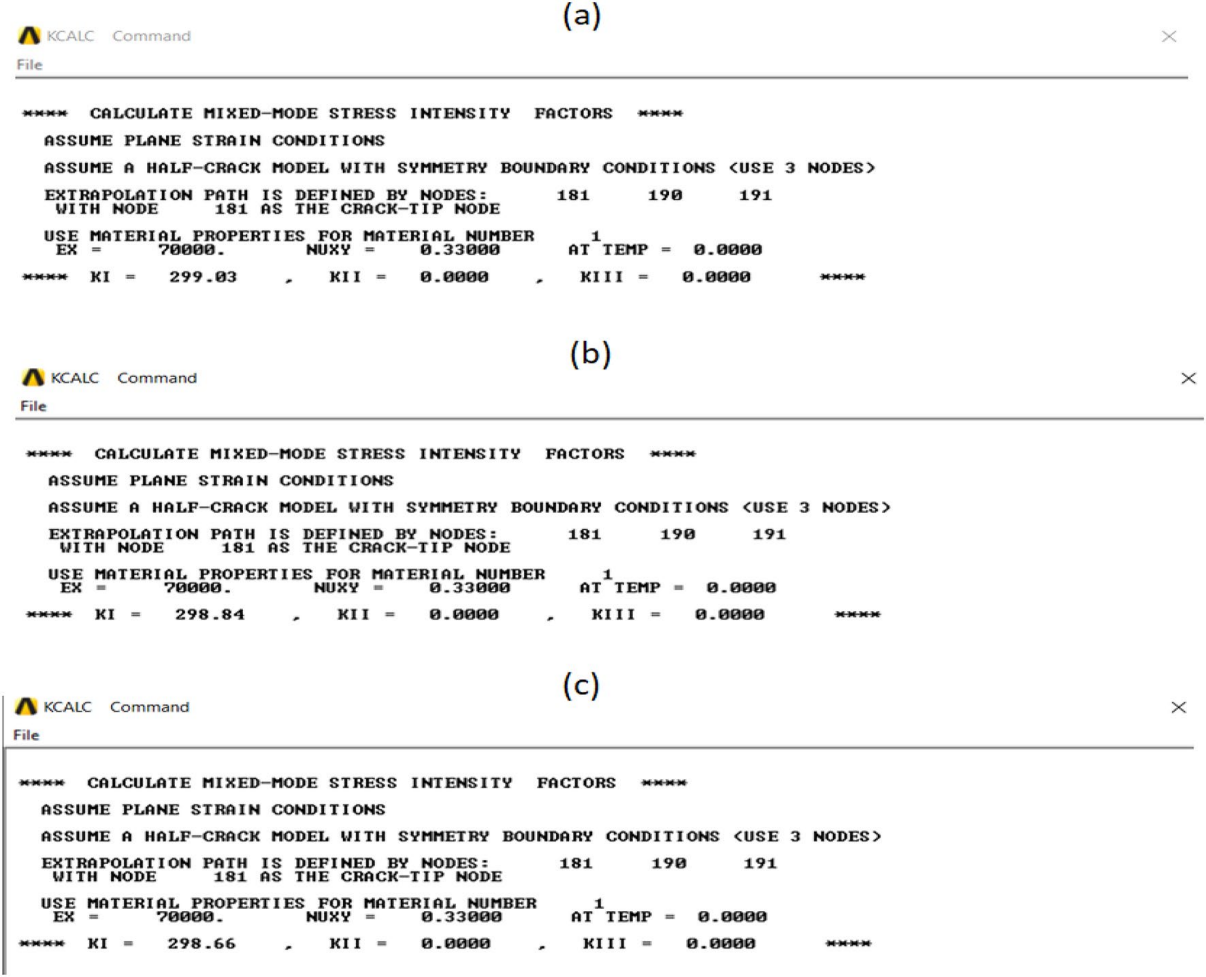
Mode I stress intensity factor (KI) with different number of singular elements: (a) 12 singular elements; (b) 14 singular elements; (c) 16 singular elements.

**Fig. 8:**
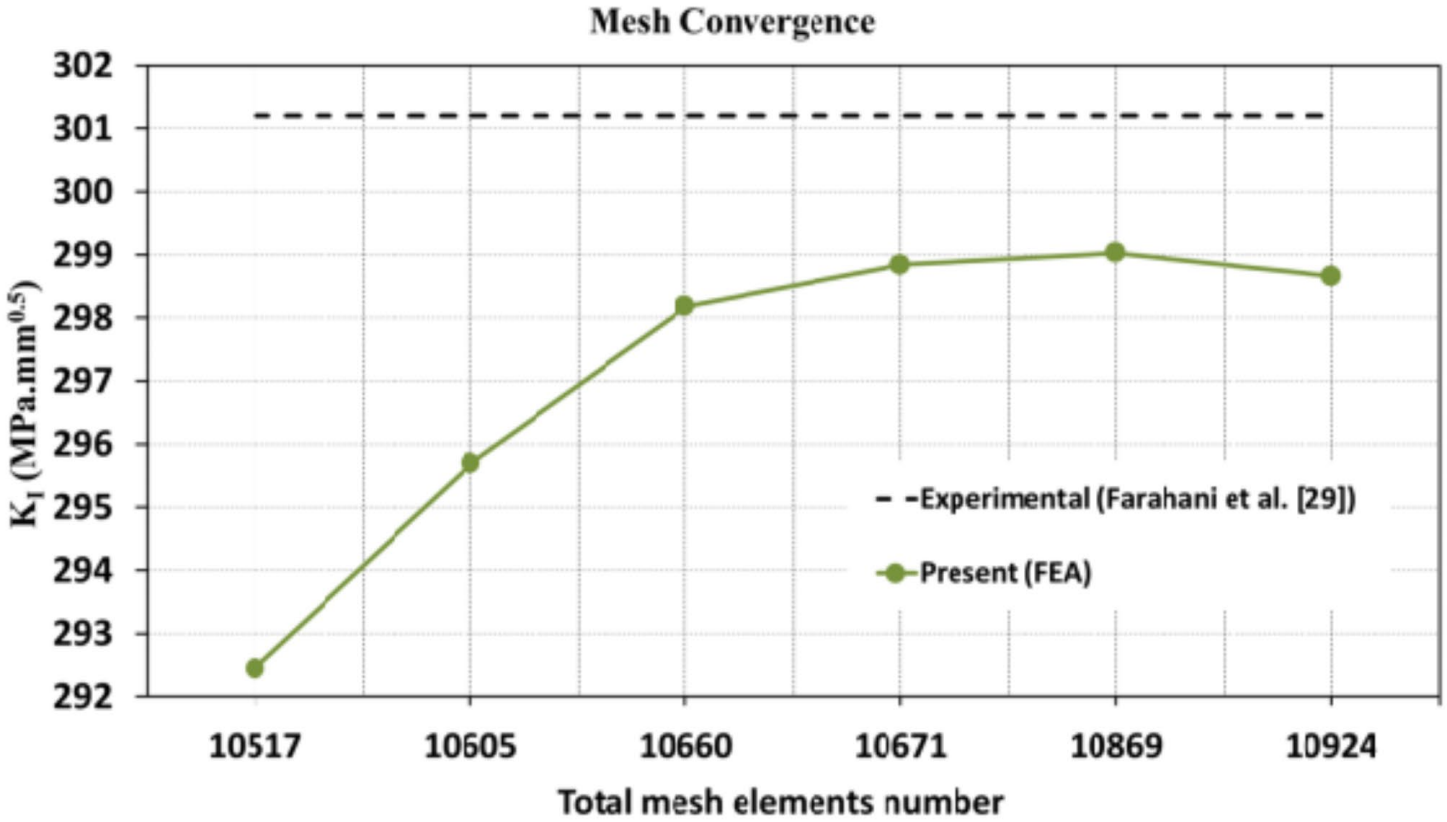
Plot of mesh convergence.

**Fig. 9:**
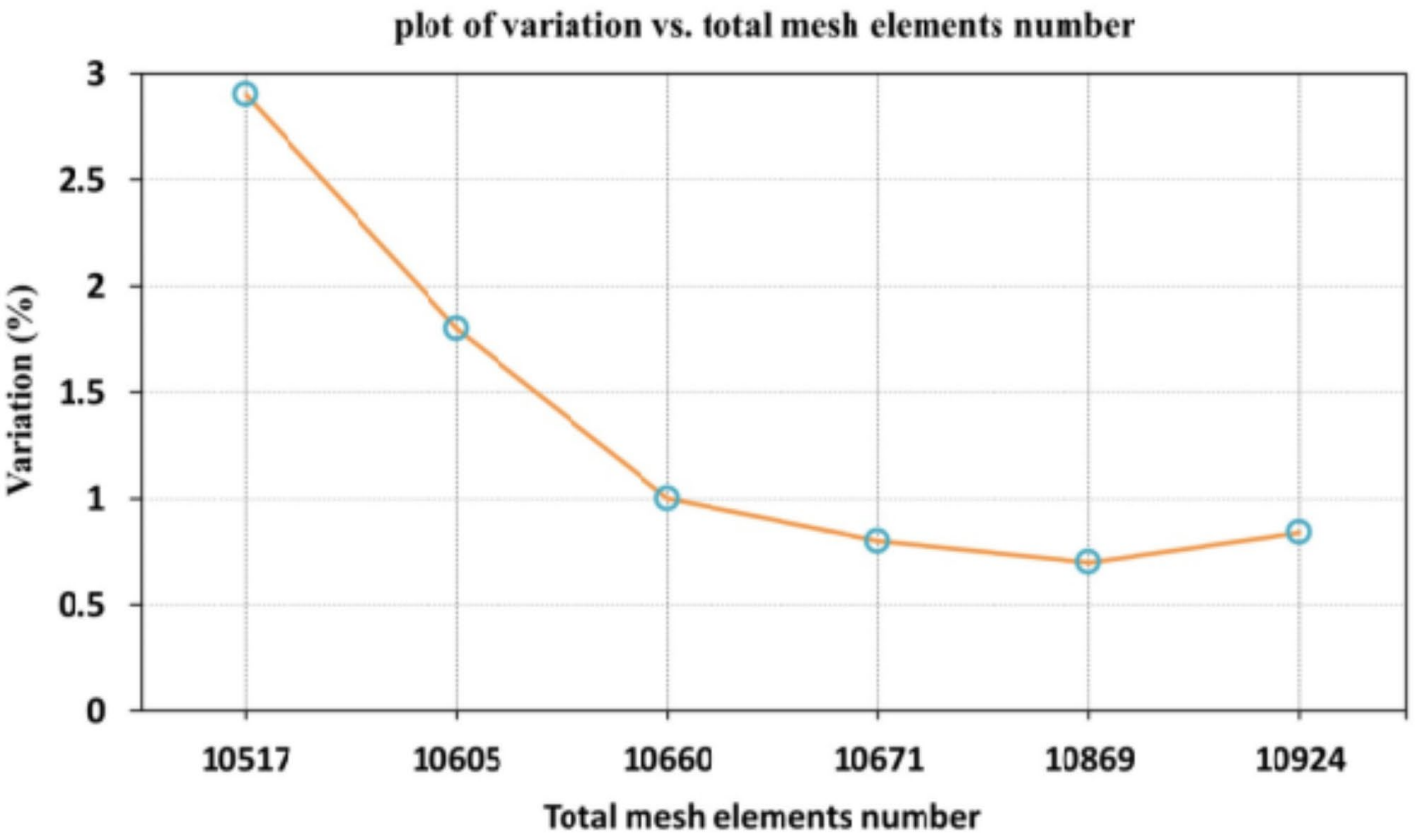
Plot of variation for K_I_ versus total mesh elements number.

When conducting finite element analysis (FEA), the meshing of the elements has a great influence on the accuracy of the results. To adapt the singularity of the crack tip field, degenerate singular elements are employed at the crack tip^28^. The geometry of the compact tension CT specimen under this study as depicted in Fig. 2 is considered as reported in^29^. To verify the accuracy of the present FEA results, FEA for compact tension CT specimen^29^ is performed in ANSYS. The material properties of the compact tension CT specimen are considered as mentioned in^29^ for validation of the present FEA results. Due to symmetry, only a half of the compact tension CT specimen was modeled in the FEA. During the meshing in FEA, focus on the zone around the crack tip must be taken into account to generate the singularity in the stresses and strains at the crack tip^30,31^. In this study, singular elements are employed to mesh the zone around the crack tip, but irregular elements are utilized for the mesh of the remaining zone of the CT specimen. The mesh generation of the 2D fracture model is created using the element type of Solid Quad 4-node 182, and the creation of a concentrated keypoint at the crack tip has been implemented. The created 2D model is extruded to build the 3D finite element mesh of the fracture model using Solid Brick 8-node 185 element type. Symmetry boundary conditions of the half compact tension CT specimen model are applied. In the considered meshed half fracture model, the center point of the hole was kinematically coupled with the hole, and the force P was applied perpendicular to the crack plane on the center point of the hole^32^. The independence solution from the mesh is investigated to identify the optimal number of singular elements at the crack tip during mesh generation. For this reason, the values of Mode I stress intensity factor K_I_ were computed using FEA at the crack tip by altering the number of singular elements at the crack tip (6, 8, 10, 12, 14, 16), as depicted in Fig. 3. The finite element mesh of the half CT specimen with 12 singular elements at the crack tip is depicted in Fig. 4. For the half CT specimen, the boundary conditions and point of imposed applied load are shown in Fig. 5. The FEA results of K_I_ are compared with experimental results of K_I_ published by Farahani et al.^29^ to assess convergence and precision of the finite element simulations in this present study. FEA results for K_I_ are indicated in Figs. 6 and 7. As shown in Table 1, it can be found that the obtained results of FEA K_I_ meet with excellent agreement with the results of experimental K_I_ already published by Farahani et al.^29^ and thus validate the precision of the FE method used in this study. From the results in Table 1, it can be seen that when the number of singular elements at the crack tip is 12, the FEA K_I_ is 299.03 MPa.mm^0.5^ including the percentage variation of about 0.7% between experimental K_I_ 301.2 MPa.mm^0.5^^29^. Figures 8 and 9 indicate the plot of mesh convergence and variation for K_I_ for different number of total mesh elements. It is noticed from Fig. 8, that the FEA results of K_I_ converges to experimental value of K_I_^29^ as number of elements increases until 10,869 elements and then decreases. Figure 9 showed that, the optimum total mesh elements number is 10,869 elements where it is exhibited minimum variation for K_I_. Therefore, it can be concluded that the optimal number of total mesh elements and singular elements at the crack tip respectively are 10,869 elements and 12 singular elements respectively to conduct accurate FEA calculations in the present study. Also the optimal number of total mesh nodes used in the finite analysis is11159 nodes. Moreover, only a model of the half compact tension CT specimen due to symmetry is used for the analysis in the present study to save time of mesh generation.

**Table 1:** Comparison of results of K_I_.

No.	Crack length (mm)	Number of singular elements	Mode I stress intensity factor K_I_ (MPa.mm^0.5^)		Variation (%)
			Farahani et al.^29^ (Experimental)	Present (FEA)	
1	14.07	6	301.2	292.44	2.9
2	14.07	8	301.2	295.69	1.8
3	14.07	10	301.2	298.18	1
4	14.07	12	301.2	299.03	0.7
5	14.07	14	301.2	298.84	0.8
6	14.07	16	301.2	298.66	0.84

## Methodology

In the present work, finite element analysis (FEA) using ANSYS is performed on a compact tension CT specimen^29^ of width (H) = 40 mm and specimen thickness (B) = 2 mm to study the Mode I stress intensity factor K_I_ at the crack tip for the compact tension CT specimen. The range of reinforcement volume fraction used for fabricate Metal-matrix composite MMC during stir casting technique is up 30%^33^. On the other hand, when the reinforcement volume fraction is very high, e.g., at 30 vol%, it will cause a serious problem of local reinforcement particle clustering^33^. In the present study the selection of percentage of SiC is reasonable value and lies in the permissible range to study the effect of volume fraction of SiC particles. For this reason, FEA was conducted on the compact tension CT specimen made from Al6061-SiC composite with volume fractions of SiC particles of 4 vol%, 6 vol%, 10 vol%, 12 vol%, and 14 vol%. The variation of Mode I stress intensity factor K_I_ at the crack tip is investigated for three different crack length-to-width (a/H) ratios, i.e., 0.35, 0.43, and 0.5. Moreover, these crack size (a/H) ratios are selected where they satisfy the condition of (a/H) ≥  0.2^29^. The material properties of aluminum 6061 (Al6061) and silicon carbide (SiC) are indicated in Table 2. To eliminate the bubbles and minimize the porosity, vigorous stirring is considered in stir casting process and this results in increase of Young’s modulus of Metal-matrix composite MMC^33^. Bhushan and Kumar^33^ prepared test specimens made of aluminum (7075 Al) matrix reinforced with 10% SiC particles using stir casting process. By stirring using mechanical stirrer for ten minutes, they obtained better distribution of SiC particles in the molten metal and near-perfect (mechanically) contacts between the matrix and the SiC particles. They found out average analytical value of Young’s modulus during the experiments. Also, they obtained theoretical value of Young’s modulus using Hashin-Shtrikman equation and comparison between theoretical and analytical value of Young’s modulus for composite exhibited difference variation of 16.18%. Thus, Hashin-Shtrikman equation Eq. 21^33,34^ is considered in the present study to calculate Young’s modulus of the Al6061-SiC composites.

Hashin-Shtrikman equation can be expressed as^33,34^:

21 $$E_{C} = \frac{{E_{m} \left[ {E_{m} \left( {1 - \beta } \right) + E_{r} \left( {\beta + 1} \right)} \right]}}{{E_{r} \left( {1 - \beta } \right) + E_{m} \left( {\beta + 1} \right)}}$$

Where E_C_ is Young’s modulus for composite, E_m_ is Young’s modulus for matrix, E_r_ is Young’s modulus for reinforcement and β is reinforcement particle concentration in the composite.

The Poisson ratio of the Al6061-SiC composites used for this study is calculated using Eqs. 22–26^35^.

Poisson ratio for composite can be written as^35^:

22 $$\nu_{C} = \frac{{3K_{C} - 2G_{C} }}{{2\left( {3K_{C} + G_{C} } \right)}}$$

Where ν_C_ is Poisson ratio for composite, K_C_ is bulk modulus for composite and G_C_ is shear modulus for composite.

Bulk modulus for composite can be written as^35^:

23 $$K_{C} = K_{m} + \left( {K_{r} - K_{m} } \right)\frac{{a\beta K_{m} }}{{\left( {1 - \beta } \right)K_{r} + a\beta K_{m} }}$$

Where K_C_ is bulk modulus for composite, K_m_ is bulk modulus for matrix and K_r_ is bulk modulus for reinforcement.

Shear modulus for composite can be written as^35^:

24 $$G_{C} = G_{m} + \left( {G_{r} - G_{m} } \right)\frac{{b\beta G_{m} }}{{\left( {1 - \beta } \right)G_{r} + b\beta G_{m} }}$$

Where G_C_ is bulk modulus for composite, G_m_ is bulk modulus for matrix and G_r_ is bulk modulus for reinforcement.

Parameter, a can be expressed as^35^:

25 $$a = \frac{{K_{r} \left( {3K_{m} + 4G_{m} } \right)}}{{K_{m} \left( {3K_{r} + 4G_{m} } \right)}}$$

Parameter, b can be expressed as^35^:

26 $$b = \frac{{G_{r} \left[ {6G_{m} \left( {K_{m} + 2G_{m} } \right) + G_{m} \left( {9K_{m} + 8G_{m} } \right)} \right]}}{{G_{m} \left[ {6G_{r} \left( {K_{m} + 2G_{m} } \right) + G_{m} \left( {9K_{m} + 8G_{m} } \right)} \right]}}$$

**Fig. 10:**
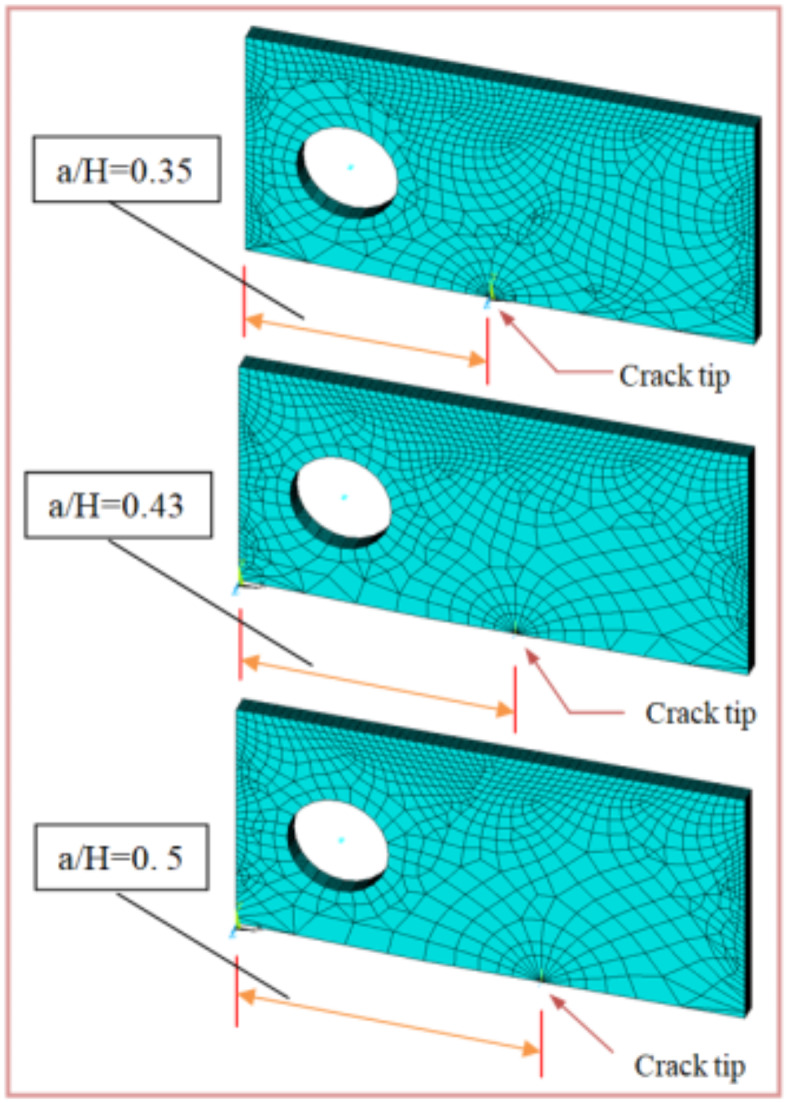
3D FE mesh of compact tension CT specimen for three different crack configurations.

**Fig. 11:**
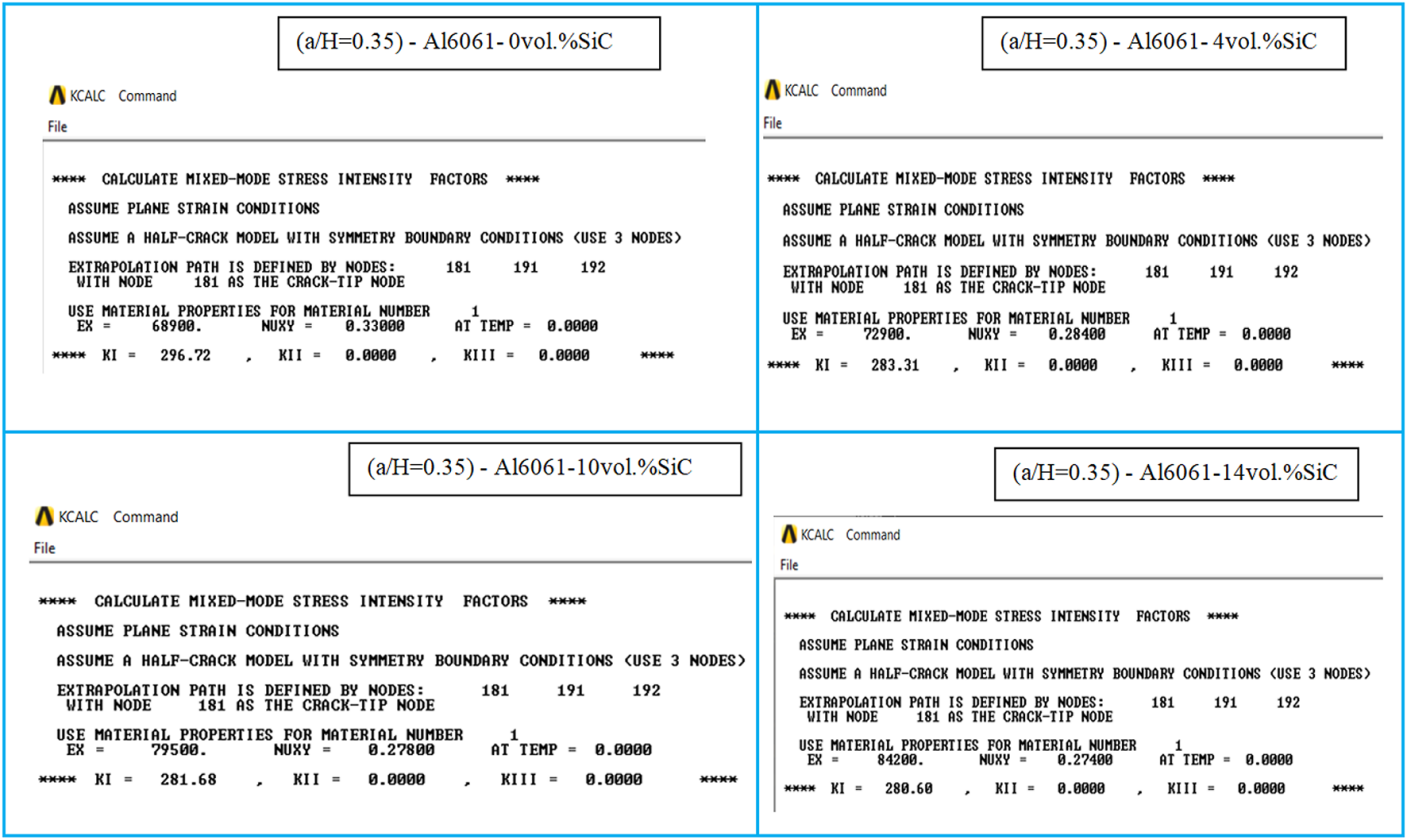
FEA results of Mode I stress intensity factor for compact tension CT specimen: (a/H) = 0.35.

**Fig. 12:**
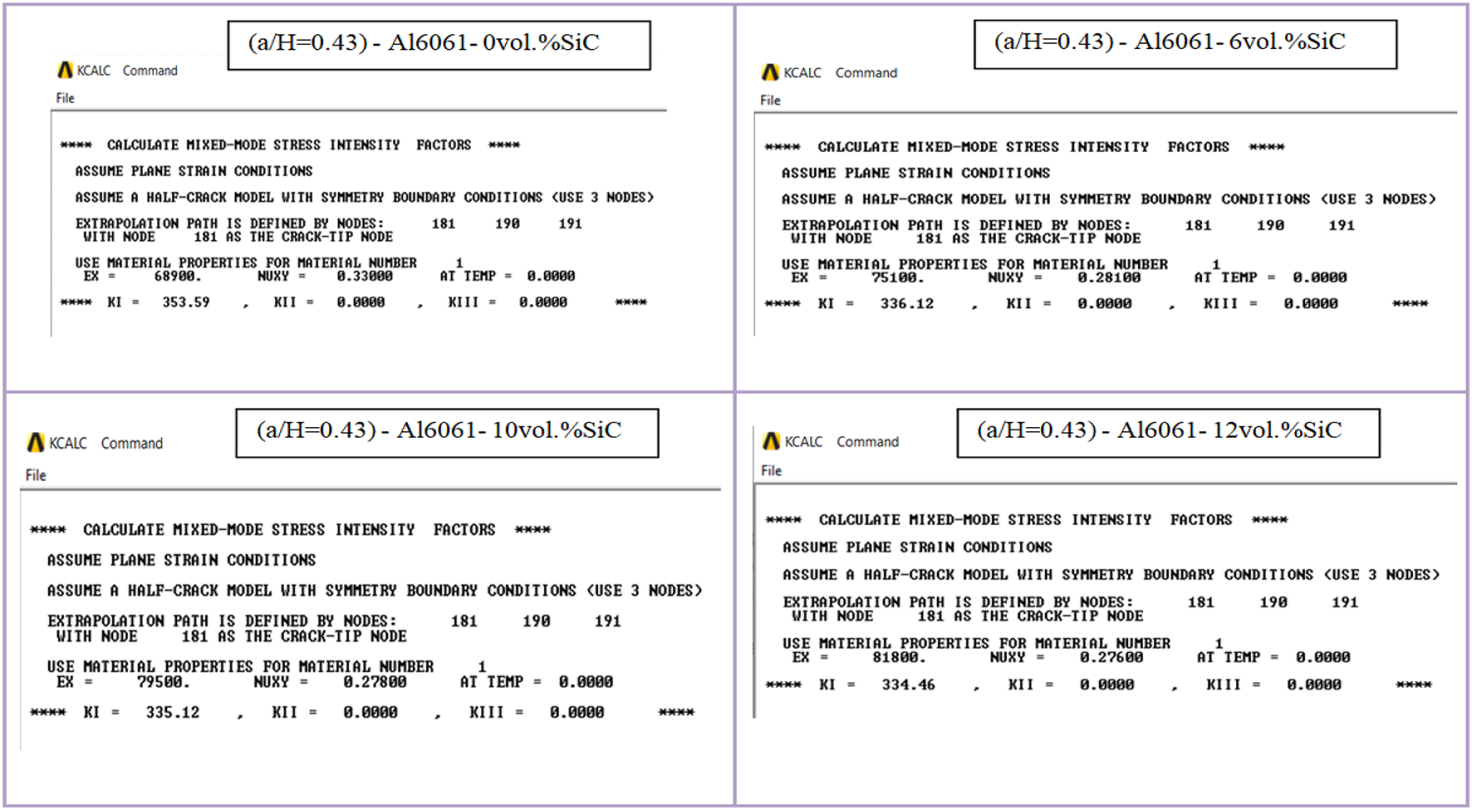
FEA results of Mode I stress intensity factor for compact tension CT specimen: (a/H) = 0.43.

**Fig. 13:**
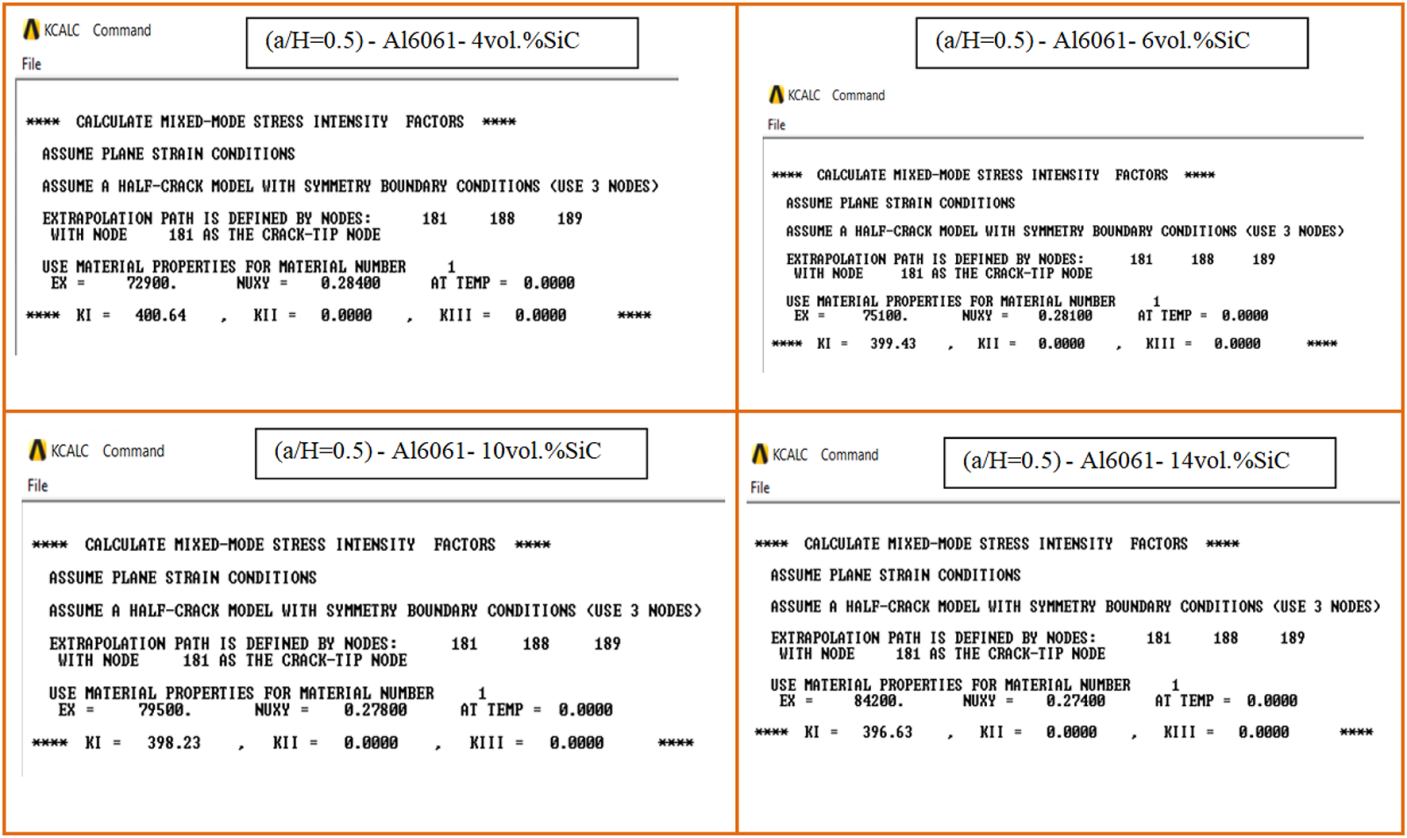
FEA results of Mode I stress intensity factor for compact tension CT specimen: (a/H) = 0.5.

The calculated mechanical properties of the Al6061-SiC composites are listed in Table 3 where Young’s modulus and Poisson ratio for composite were considered as material properties in the finite element analysis. In FEA, only half of the compact tension specimen with symmetry boundary conditions is considered and investigated under the plane strain condition. Two element types, Plane182 and Solid185, are employed, and the mesh is then generated with 12 singular elements at the crack tip. The load of a magnitude *P* = 603 N was applied perpendicular to the crack plane on the center point of the hole. 3D FE mesh of compact tension CT specimen for three different crack configurations (a/H = 0.35, a/H = 0.43, and a/H = 0.5) is depicted in Fig. 10. The crack model is created, and the plane strain Mode I stress intensity factor K_I_ has been determined using the KCALC command. The FEA results of Mode I stress intensity factor are indicated in Figs. 11, 12 and 13; Table 4.

**Table 2:** Mechanical properties of Al6061 and Silicon carbide (SiC).

Young’s modulus (GPa)		Poisson’s ratio		Shear modulus (GPa)		Bulk modulus (GPa)	
Al6061	SiC^36^	Al6061	SiC^36^	Al6061	SiC	Al6061	SiC
68.9	410	0.33	0.14	25.9	179.8	52.2	189.8

**Table 3:** Mechanical properties of Al6061-SiC composite.

No.	Composite MMC	Young’s modulus, E_C_ (GPa)	Bulk modulus, K_C_ (GPa)	Shear modulus, G_C_ (GPa)	Poisson’s ratio, ν_C_
1	Al6061-4vol.%SiC	72.9	54.4	27.5	0.284
2	Al6061-6vol.%SiC	75.1	55.5	28.4	0.281
3	Al6061-10vol.%SiC	79.5	57.9	30.2	0.278
4	Al6061-12vol.%SiC	81.8	59.1	31.2	0.276
5	Al6061-14vol.%SiC	84.2	60.4	32.1	0.274

**Table 4:** FEA results of Mode I stress intensity factor for a MMC composite CT specimen.

No.	MMC composite specimen	Mode I stress intensity factor K_I_ (MPa.mm^0.5^)		
		(a/H = 0.35)	(a/H = 0.43)	(a/H = 0.5)
1	Al6061-0vol.%SiC	296.72	353.59	420.49
2	Al6061-4vol.%SiC	283.31	337.13	400.64
3	Al6061-6vol.%SiC	282.49	336.12	399.43
4	Al6061-10vol.%SiC	281.68	335.12	398.23
5	Al6061-12vol.%SiC	281.14	334.46	397.43
6	Al6061-14vol.%SiC	280.6	333.8	396.63

### T-stress

One of the most important parameters, named T-stress, is considered to investigate the material behavior with the accuracy of failure prediction^37^. The polar angle θ effects the stress distributions ahead of a crack tip^38^, where the T-stress for θ = 0 can be expressed as the difference of the normal stresses^39^:

27 $$T = \sigma_{xx} - \sigma_{yy}$$

Where σ_xx_ is the stress parallel to the crack and σ_yy_ is the opening stress.

To adequately describe the stress state surrounding the crack, it is crucial to include an additional non-singular term, i.e., the T-stress, in the Williams expansion^40^. The size and shape of the plane strain crack tip plastic zone depend on the sign and magnitude of the T-stress^41^. The sign and magnitude of the T-stress basically affect the level of crack-tip stress triaxiality^42^. High crack-tip constraint, i.e., strengthening the level of crack-tip stress tri-axiality, is due to positive T-stress, but loss of crack-tip constraint, i.e., reduction of the level of crack-tip stress tri-axiality, is due to negative T-stress^42^. Fracture toughness, crack angle, and the stability of crack growth can be influenced by the positive or negative numerical magnitude of T-stress^43^. Kudari and Kodancha^18^ inferred from their study that T_11_-stress is not a constant through the thickness of the CT specimen along the crack front, where it is a maximum at the center of the specimen than at the surface. In this work, the magnitudes of T_11_-stress and T_33_-stress, respectively, at the center of the compact tension CT specimen along the crack front are computed using Eq. 28^18^ and Eq. 29^44^, respectively.

T_11_-stress at the center of the compact tension CT specimen along the crack front can be expressed as^18^:

28 $$\frac{{T_{11 - \max } }}{{\left( {\frac{{K_{I - \max } }}{{\sqrt {\pi B} }}} \right)}} = 0.1477 + 0.93746\left( \frac{B}{H} \right) - 0.87183\left( \frac{B}{H} \right)^{2} + 0.35186\left( \frac{B}{H} \right)^{3}$$

T_33_-stress at the center of the compact tension CT specimen along the crack front can be expressed as^44^:

29 $$T_{33} = E\varepsilon_{33} + \nu T_{11}$$

**Fig. 14:**
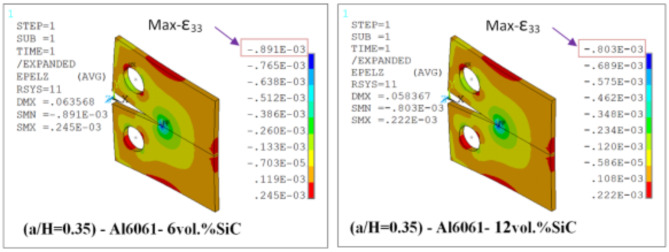
FEA contours of Z-component of strain of CT specimen (a/H) = 0.35: reflect about XZ of cyclic symmetry.

**Fig. 15:**
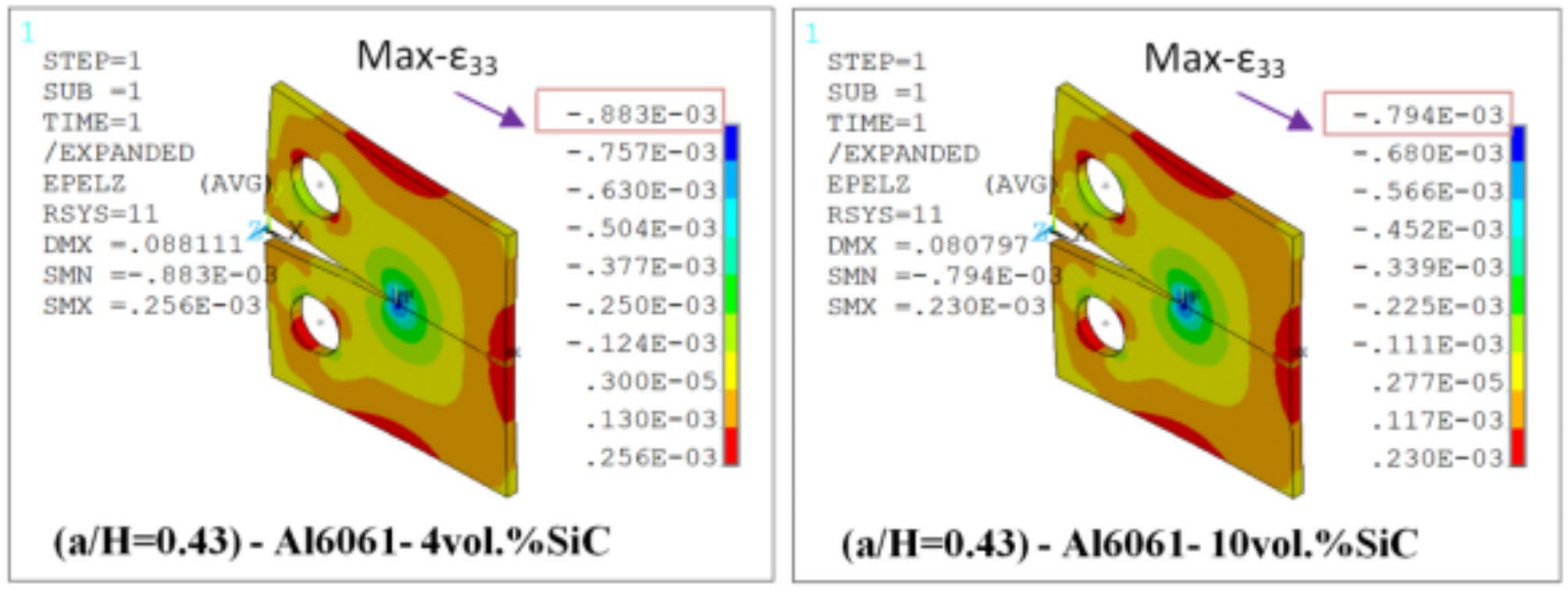
FEA contours of Z-component of strain of compact tension CT specimen (a/H) = 0.43: reflect about XZ of cyclic symmetry.

**Fig. 16:**
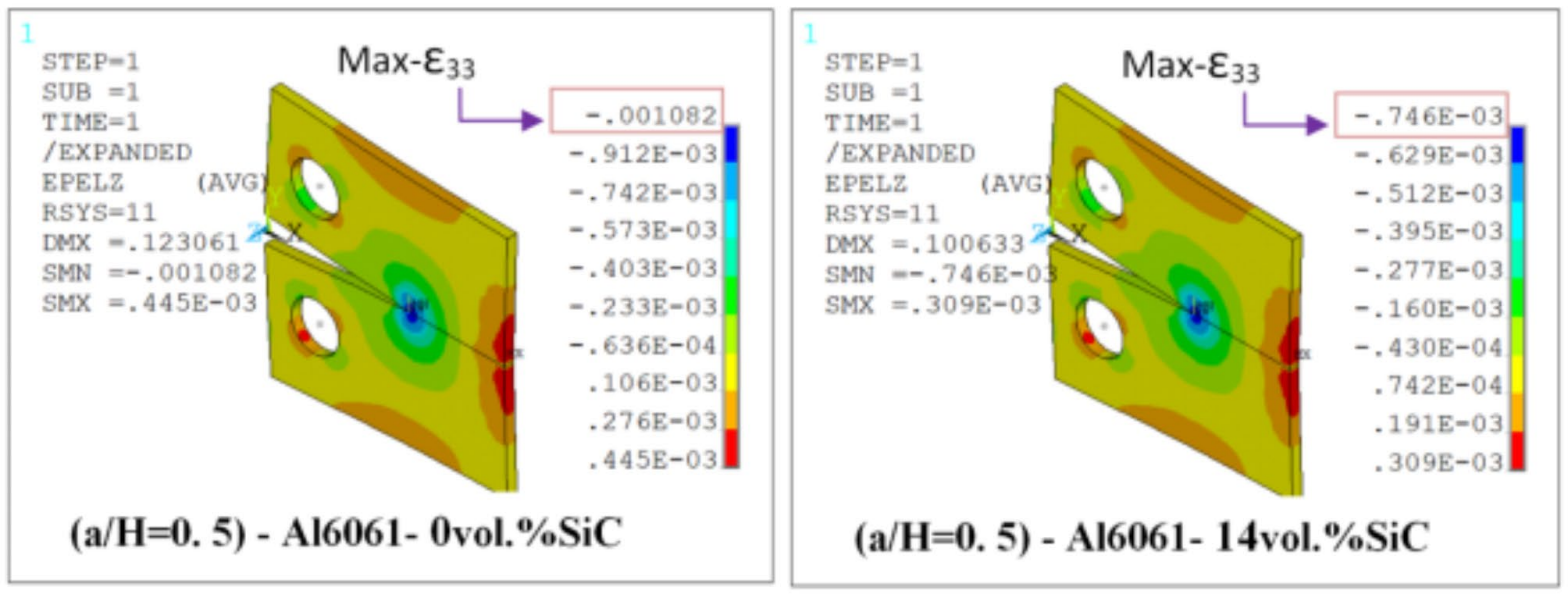
FEA contours of Z-component of strain of compact tension CT specimen (a/H) = 0.5: reflect about XZ of cyclic symmetry.

**Fig. 17:**
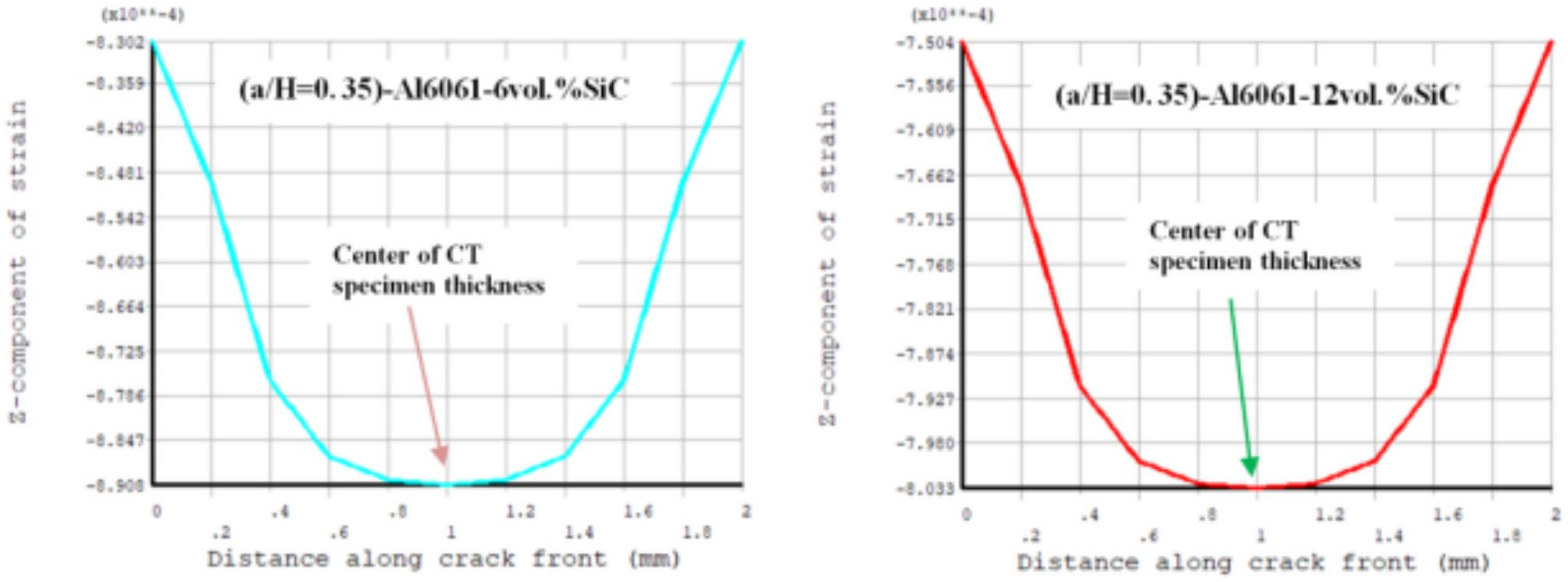
FEA result of Z-component of strain through a thickness of the CT specimen along crack font (a/H) = 0.35.

**Fig. 18:**
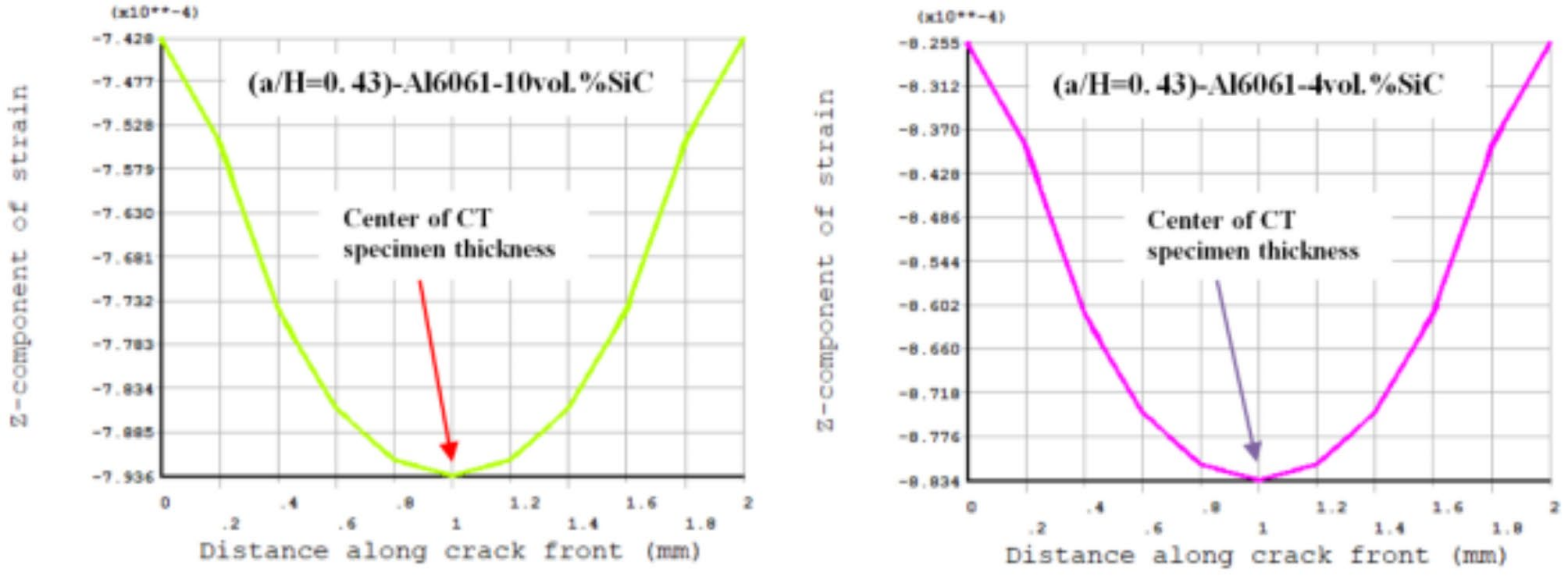
FEA result of Z-component of strain through a thickness of the CT specimen along crack font (a/H) =0.43

**Fig. 19:**
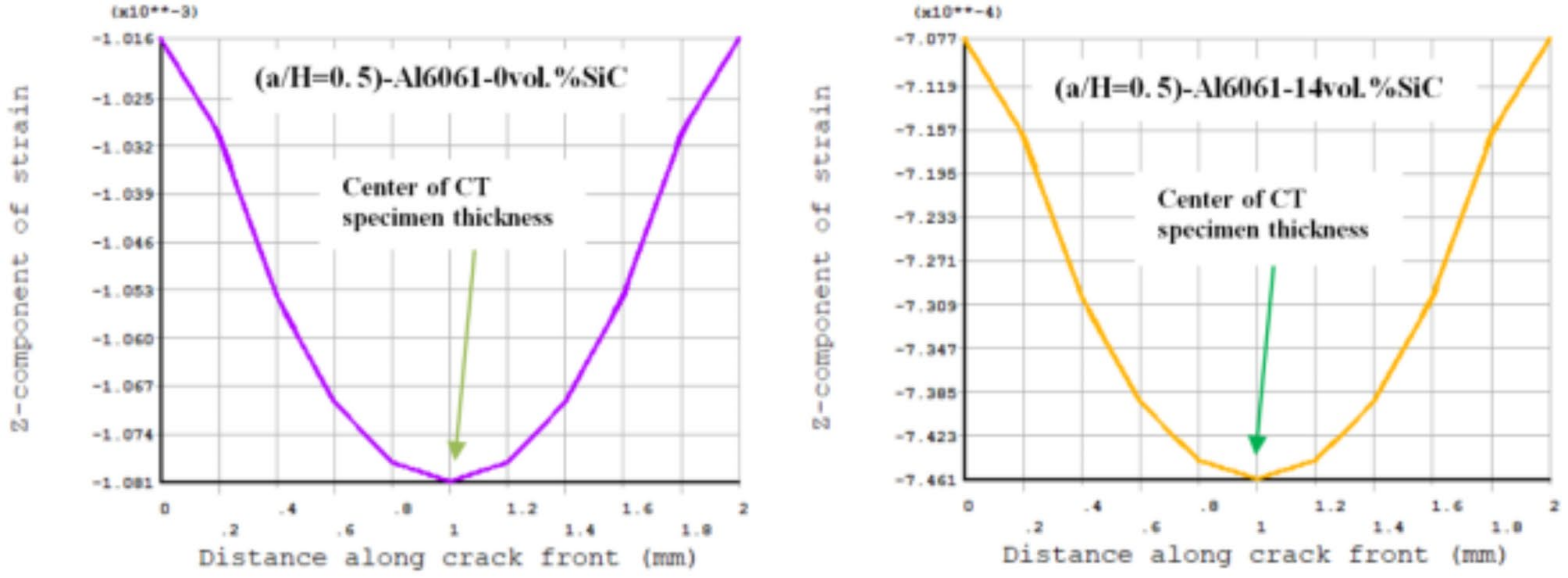
FEA result of Z-component of strain through a thickness of the CT specimen along crack font (a/H) = 0.5.

Results of T_11_-stress at the center of the compact tension CT specimen along the crack front have been listed in Table 5. The ε_33_ (strain) is extracted from finite element analysis (FEA), and some FEA results of strain ε_33_ are shown in Figs. 14, 15, 16, 17, 18 and 19.

FEA results of strain ε_33_ and T_33_-stress are given in Table 6.

**Table 5:** FEA results of T_11_ for a composite CT specimen.

No.	Composite CT specimen	T_11_-max (MPa)		
		(a/H = 0.35)	(a/H = 0.43)	(a/H = 0.5)
1	Al6061-0vol.%SiC	22.75	27.1	32.24
2	Al6061-4vol.%SiC	21.72	25.85	30.72
3	Al6061-6vol.%SiC	21.66	25.77	30.62
4	Al6061-10vol.%SiC	21.6	25.69	30.53
5	Al6061-12vol.%SiC	21.55	25.64	30.47
6	Al6061-14vol.%SiC	21.51	25.59	30.41

**Table 6:** FEA results of ε_33_ and T_33_ for a composite compact tension CT specimen.

No.	Composite CT specimen	(a/H = 0.35)		(a/H = 0.43)		(a/H = 0.5)	
		ε_33_ (FEA)	T_33_-max (MPa)	ε_33_ (FEA)	T_33_-max (MPa)	ε_33_ (FEA)	T_33_-max (MPa)
1	Al6061-0vol.%SiC	0.001137	85.85	0.001075	83.01	0.001082	85.19
2	Al6061-4vol.%SiC	0.928 × 10^−3^	73.82	0.883 × 10^−3^	71.71	0.891 × 10^−3^	73.68
3	Al6061-6vol.%SiC	0.891 × 10^−3^	73	0.849 × 10^−3^	71	0.857 × 10^−3^	72.96
4	Al6061-10vol.%SiC	0.833 × 10^−3^	72.23	0.794 × 10^−3^	70.26	0.801 × 10^−3^	72.17
5	Al6061-12vol.%SiC	0.803 × 10^−3^	71.63	0.766 × 10^−3^	69.74	0.773 × 10^−3^	71.64
6	Al6061-14vol.%SiC	0.775 × 10^−3^	71.15	0.739 × 10^−3^	69.24	0.746 × 10^−3^	71.15

## Results and discussion

**Fig. 20:**
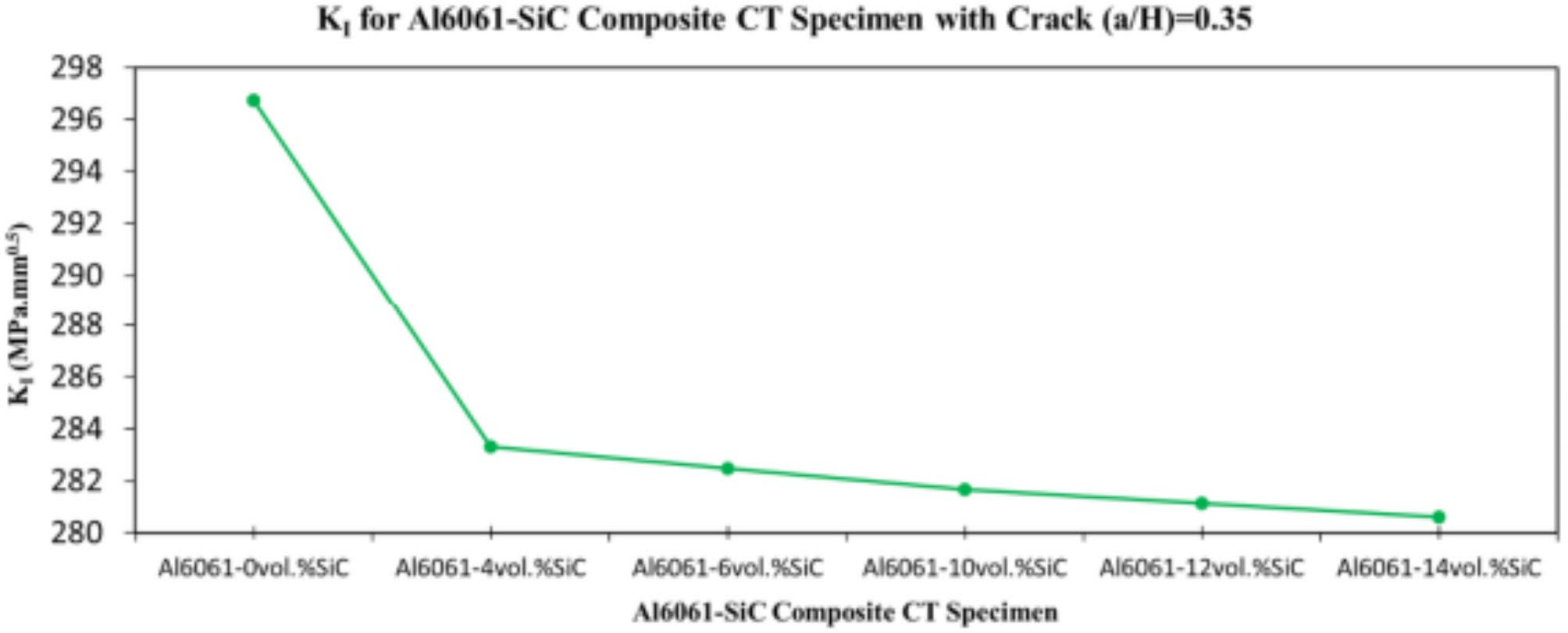
Variation of stress intensity factor at crack tip for composite CT specimen with different vol% SiC particles for (a/H) = 0.35.

**Fig. 21:**
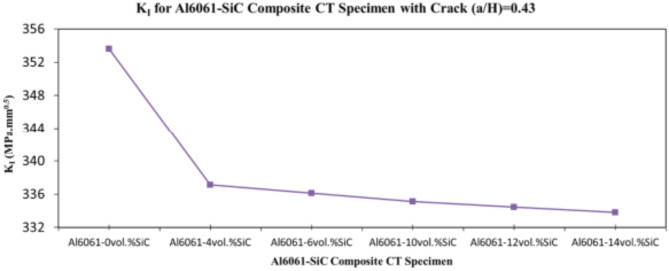
Variation of stress intensity factor at crack tip for composite CT specimen with different vol% SiC particles for (a/H) = 0.43.

**Fig. 22:**
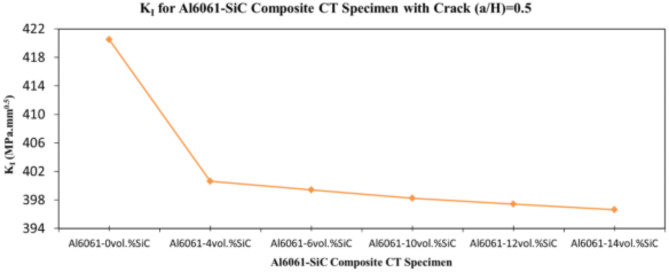
Variation of stress intensity factor at crack tip for composite CT specimen with different vol% SiC particles for (a/H) = 0.5.

**Fig. 23:**
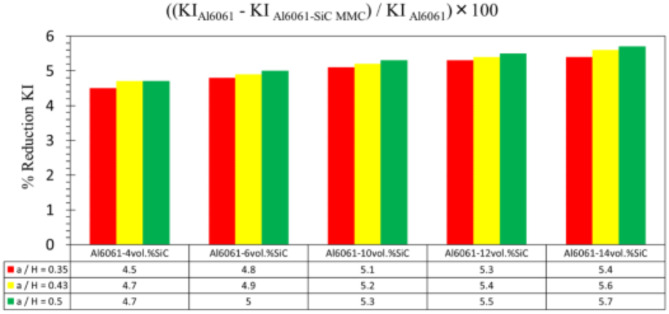
Percentage reduction of stress intensity factor for composite CT specimen.

**Fig. 24:**
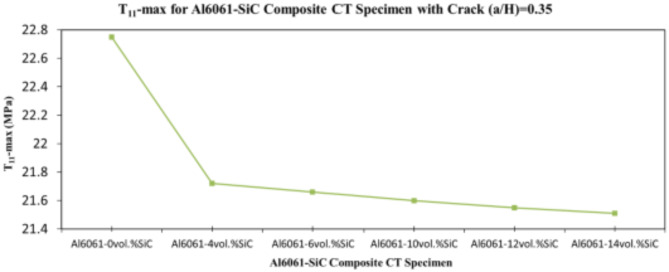
Variation of T_11_-max for composite CT specimen with different vol% SiC particles for (a/H) = 0.35.

**Fig. 25:**
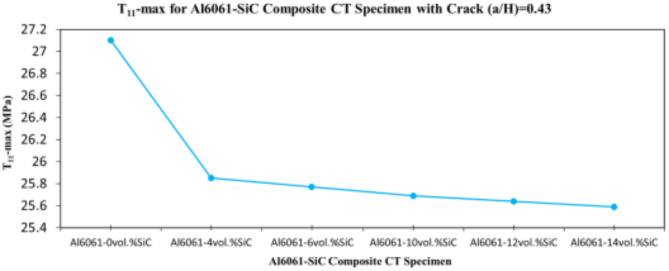
Variation of T_11_-max for composite CT specimen with different vol% SiC particles for (a/H) = 0.43.

**Fig. 26:**
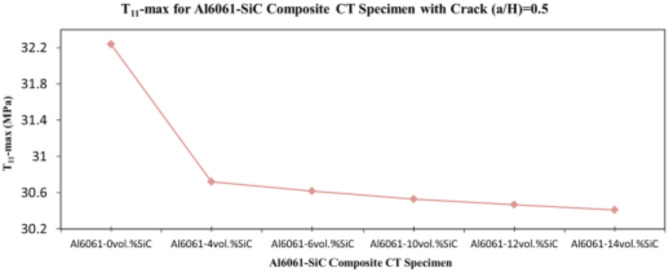
Variation of T_11_-max for composite CT specimen with different vol% SiC particles for (a/H) = 0.5.

**Fig. 27:**
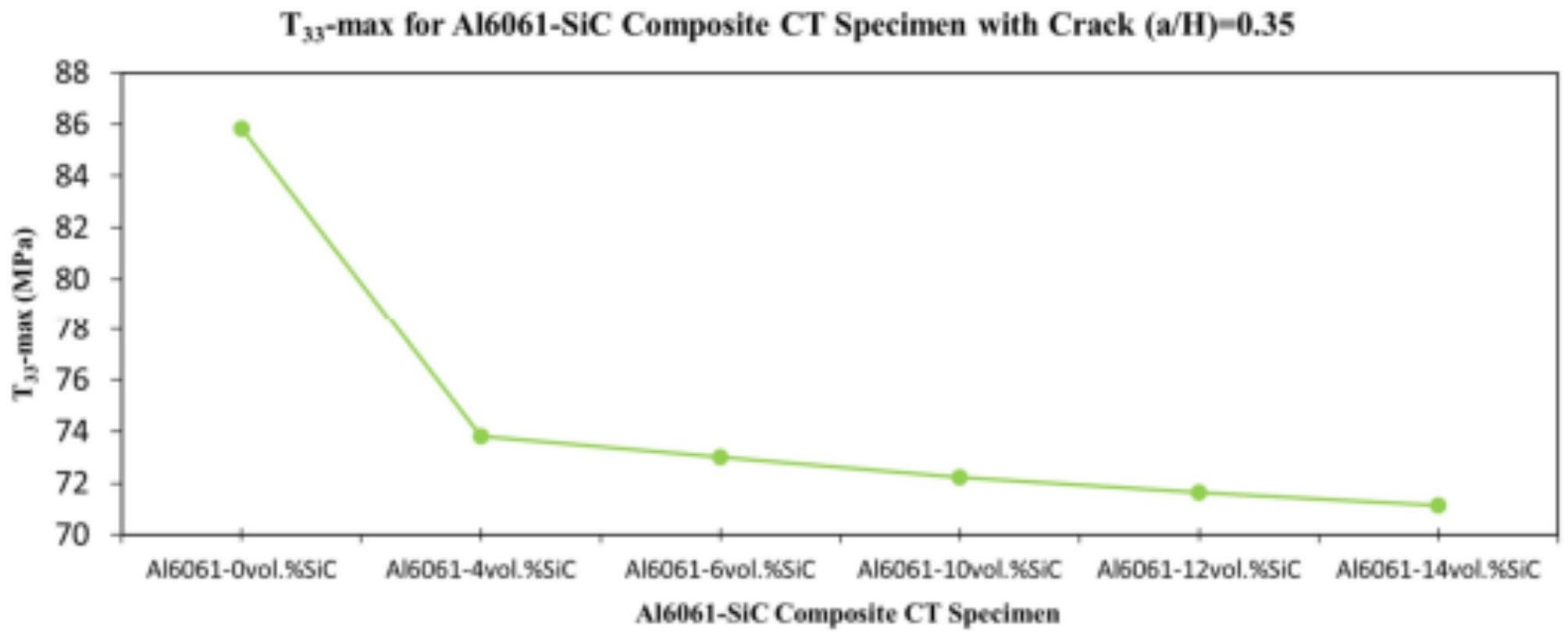
Variation of T_33_-max for composite CT specimen with different vol% SiC particles for (a/H) = 0.35.

**Fig. 28:**
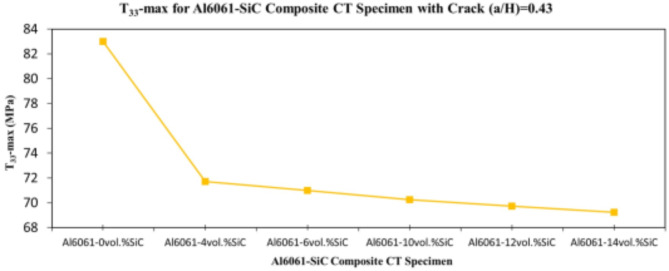
Variation of T_33_-max for composite CT specimen with different vol% SiC particles for (a/H) = 0.43.

**Fig. 29:**
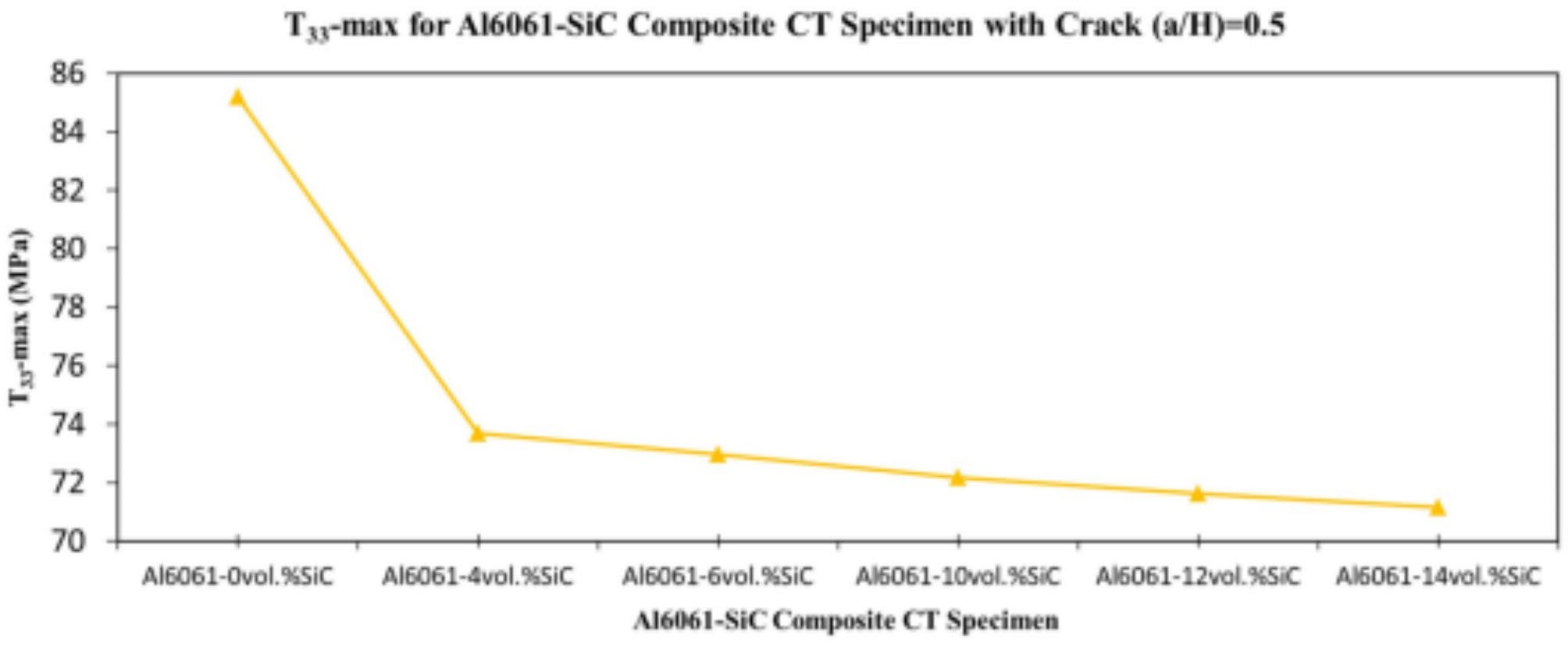
Variation of T_33_-max for composite CT specimen with different vol% SiC particles for (a/H) = 0.5.

In this section, the main extracted aspects from the study are indicated. Figures 20, 21 and 22 show variation of stress intensity factor at crack tip for Al6061-SiC composite compact tension CT specimen versus different volume fractions of SiC particles. From Figs. 20, 21 and 22, it is found that as the volume fraction of SiC increases, the stress intensity factor K_I_ for composite compact tension CT specimen decreases for (a/H) = 0.35, 0.43, and 0.5. Where the values of K_I_ for Al6061-4vol.%SiC composite CT specimen are 283.31 MPa.mm^0.5^, 337.13 MPa.mm^0.5^and 400.64 MPa.mm^0.5^ respectively, for (a/H) = 0.35, 0.43, and 0.5, respectively. Also, the values of K_I_ for Al6061-14vol.%SiC composite CT specimen are 280.6 MPa.mm^0.5^, 333.8 MPa.mm^0.5^and 396.63 MPa.mm^0.5^ respectively, for (a/H) = 0.35, 0.43, and 0.5, respectively. Figure 23 shows the percentage reduction of stress intensity factor for composite compact tension CT specimen over that of Al6061 CT specimen. As shown in Fig. 23, the percentage reduction in the K_I_ increases with increasing volume fractions of SiC particles for (a/H) = 0.35, 0.43, and 0.5. It is clearly observed from Fig. 23 that Al6061-14vol%SiC exhibits the maximum percentage reduction in the K_I_ compared to the other four compositions of Al6061-SiC composites, i.e. Al6061-4vol. %SiC, Al6061-6vol. %SiC, Al6061-10vol. %SiC and Al6061-12vol. %SiC. Al6061-14vol. %SiC composite compact tension CT specimen for (a/H) = 0.35, 0.43, and 0.5, respectively, has a percentage reduction in the K_I_ over that of Al6061 CT specimen 5.4%, 5.6%, and 5.7%, respectively. Also, Al6061-4vol.%SiC composite CT specimen for (a/H) = 0.35, 0.43, and 0.5, respectively, exhibits a percentage reduction in the K_I_ over that of Al6061 CT specimen 4.5%, 4.7%, and 4.7%, respectively. Figures 24, 25 and 26 indicate variations in the maximum values of T_11_-stress at the center of the Al6061-SiC composite CT specimen along the crack front versus different volume fractions of SiC particles. From Figs. 24, 25 and 26, it is clearly found that as the volume fraction of SiC rises, T_11_-stress reduces significantly. Al6061-14vol.%SiC composite CT specimen has lower values of T_11_-stress compared to other four compositions of Al6061-SiC composites. It is found that the values of T_11_ are 21.51 MPa, 25.59 MPa, and 30.41 MPa, respectively, for (a/H) = 0.35, 0.43, and 0.5, respectively, in the case of the Al6061-14vol.%SiC composite CT specimen. Also, in the case of the Al6061-4vol.%SiC composite CT specimen, the values of T_11_ were 21.72 MPa, 25.85 MPa, and 30.72 MPa, respectively, for (a/H) = 0.35, 0.43, and 0.5, respectively. From Figs. 27, 28 and 29, it can be found that with an increase in volume fraction of SiC, T_33_-stress of the Al6061-SiC composite compact tension CT specimen was reduced. There is a more significant decrease in the values of T_33_-stress of the Al6061-14vol%SiC composite compact tension CT specimen (71.15, 69.24 and 71.15 MPa) than those of the Al6061 CT specimen (85.85, 83.01 and 85.19 MPa), as can be seen in Figs. 27, 28 and 29.

## Conclusions

The main objective of this study is to investigate the effect of the addition of silicon carbide (SiC) particulates into the aluminum 6061 (Al6061). Finite element analysis has been implemented for the Al6061-SiC composite compact tension specimen with a volume fraction of SiC particles of 4 vol%, 6 vol%, 10 vol%, 12 vol%, and 14 vol%. The mode I stress intensity factor K_I_, T_11_-stress, and T_33_-stress are calculated for both Al6061 and Al6061-SiC composite CT specimens for a plane strain condition for various crack length to width ratios (a/H = 0.35, 0.43, and 0.5). From the FEA results presented, it is found that the variation of the volume fraction of SiC reinforcement particles with the Al6061 alloy matrix plays a vital role in the variation of the K_I_ and T-stress. When the volume fraction of SiC particles increases, then the K_I_, T_11_-stress, and T_33_-stress decrease. Also, optimum results were found for the Al6061-14vol.%SiC composite CT specimen as compared to those of aluminum Al6061. From this present study, it is inferred that the addition of SiC particulates to the Al6061 alloy enhances the fracture mechanics properties and the strength of the material and lets the material satisfy the maximum stress failure criterion.

## Data Availability

Data is provided within the manuscript. All relevant data supporting the findings of this study are included within the manuscript.
